# Supplementary material for: The aesthetic value of reef fishes is globally mismatched to their conservation priorities
Source: PLoS Biol. 2022 Jun 7;20(6):e3001640. doi: 10.1371/journal.pbio.3001640 (PMC9173608; doi:10.1371/journal.pbio.3001640)
Supplement: S1 File — Text A. Image features analysis. Text B. Image sampling strategy. Text C. Sociocultural background. Text D. Elo scores. Text E. Deep learning algorithm. Text F. Relationship between features and aesthetic values. Text G. Phylogenetic analysis. Text H. Ecological traits. Text I. Mean aesthetic value. Fig A. Representation of the three-dimensional CIELAB space. Fig B. Cluster analysis performed for Holacanthus ciliaris. Fig C. HSV color space. Fig D. Illustration of the analysis led with the Momocs package. Fig E. Projection of the 4,881 images of our collection on the 2 first axes of the PCA on the images’ features. Fig F. Questionnaire on the sociocultural background of the judges. Fig G. Number of judges per country. The map was obtained from rworldmap v1.3.6 R package, which uses Natural Earth as base layer (https://www.naturalearthdata.com/downloads/10m-physical-vectors/). Fig H. Summary of sociocultural background of the 13,000 judges. Fig I. Variation of the Elo scores of the images as matches accumulate. Fig J. Linear regression between the Elo scores for the 21 images in common between the 2 surveys. Fig K. Effect of the size of the input images on the performances of the model. Fig L. Architecture of ResNet50 modified to predict the aesthetic values. Fig M. Relationship between the Evolutionary Distinctiveness of species (in MY) and their aesthetic value. Fig N. Mean aesthetic values of families with more than 10 species presented in decreasing order. Fig O. Comparison between the aesthetic value of the fish species and their ecological traits. Fig P. Number of images per species. Fig Q. Relationship between the aesthetic values computed using the maximum and the mean values. Fig R. Phylogenetic history and ecological originality with mean aesthetic values. Fig S. Conservation status with mean aesthetic values. Table A. Analysis of deviance in the generalized linear mixed model. Table B. Pagel’s λ. Table C. List of the ecological traits used with their natur [file pbio.3001640.s001.docx]

**Supporting Information**

Global mismatch between the aesthetic value of reef fishes and their conservation priorities

Juliette Langlois, François Guilhaumon, Florian Baletaud, Nicolas Casajus, Cédric De Almeida Braga, Valentine Fleuré, Michel Kulbicki, Nicolas Loiseau, David Mouillot, Julien P. Renoult, Aliénor Stahl, Rick D. Stuart Smith, Anne-Sophie Tribot and Nicolas Mouquet *.

* Corresponding to **:** nicolas.mouquet@cnrs.fr

**This PDF file includes:**

S1 Text A to I

S1 Figs A-S

S1 Tables A-D

SI References

**All relevant code is available from the GitHub Repository:**

https://github.com/nmouquet/RLS_AESTHE

**S1 Text**

A : IMAGE FEATURES ANALYSIS

Several metrics were computed to characterize the fish images features potentially linked to aesthetic value. The choice of these metrics was based on literature review of previous works studying the aesthetic value of biodiversity [1-6]. Four different classes of metrics were selected. They respectively characterize : (a) the heterogeneity of colors, (b) the geometry of color patterns, (c) the perceptual lightness and saturation and (d) the shape of the fish body outline.

## Color heterogeneity (CL)

We based our color analysis on the K-means clustering algorithm to separate the colors in the image color space. The K-means algorithm can use a fixed or a varying number of centroids and then allocates every pixels to the nearest cluster. To characterize each pixel of an image, we used the CIELAB color space (Fig A) which expresses colors with three values: *L** for lightness, *a** and *b** for the four unique colors of human vision: red, green, blue, and yellow (Fig A). The CIELAB color space is perceptually uniform, which explains why this color space is frequently used to model human color vision.

Rather than letting the K-means algorithm select the best number of clusters for each image (which would generate a different number of clusters for each image) we fixed the number of clusters to 9 for all images. This value was chosen in preliminary analyses performed with the images available in Tribot et al. [7] where we found that the correlation between the color metrics and the aesthetic value of fishes was higher with 9 clusters than with less clusters (more clusters did not increase the correlation further). Fig B shows an example of the cluster analysis performed for *Holacanthus ciliaris*.

The position of the cluster centers on the a*b* color space was used to compute metrics of color heterogeneity: CL_cie_d_mean and CL_cie_d_sd, which respectively measure the mean distance between all cluster centers and its standard deviation. CL_hullarea measures the area of the convex hull (the smallest convex shape enclosing all cluster’s center points in the a*b* CIELAB color space). The higher these values, the more heterogeneous and distant the colors of the different clusters of an image.

More information on the CIELAB space can be found here :

<https://en.wikipedia.org/wiki/CIELAB_color_space>

<https://hiweller.github.io/colordistance/color-spaces.html>

Here we summarize the 3 variables measured to characterize the image’s color heterogeneity :

| CL_cie_d_mean | mean distance between all clusters centers points on the a*b* CIELAB color space. |
| --- | --- |
| CL_cie_d_sd | standard deviation of the CL_cie_d_mean. |
| CL_hullarea | area of the convex hull (the smallest convex shape enclosing all cluster center points on the a*b* CIELAB color space). |

## Geometry of color patterns (SH)

We measured various statistics on the shape of each color cluster produced by the K-means algorithm (see above). These metrics were computed with the *PatchStat* function of the *SDMTools* *v1.1-221.2* R package (<https://www.rforge.net/SDMTools/>) based on *fragstats* [8]. For these shape metrics, the standard deviation is also meaningful as it allows to differentiate images containing contrasted patches from images containing similar patches in size and shape.

Here, we summarize the 4 variables measured to characterize the cluster’s shape:

| SH_n.core.cell (mean and sd) | The number of cells in the core area of the cluster, without the edge area. |
| --- | --- |
| SH_perimeter (mean and sd) | Perimeter of the cluster including any internal holes. |
| SH_perim.area.ratio (mean and sd) | The ratio of the cluster perimeter to area. |
| SH_core.area.index (mean and sd) | Proportion of the total area occupied by the core cells. |

## Lightness and saturation (LS)

We used the HSV (Hue, Saturation, Value) color space that differentiates saturation (S) from perceptual lightness (V). The HSV space, also known as the HSB (Hue, Saturation, Brightness) space, is derived from the RGB (Red, Green, Blue) space and is considered as a good perceptual color model. The HSV space can be represented as a cylinder (Fig C) where hue (H) is the azimuth, each angle corresponding to a segmented color, value (V), on the central axis is the height and ranges from 0 for black at the bottom to 1 for white at the top (hereafter called lightness) and saturation (S) is the radial distance. Also ranging from 0 at the center to 1 at the edge, S represents the intensity of the color compared to the maximum possible intensity. As color (hue) was already studied with the CIELAB color space analysis (see above), attention is here focused on saturation (S) and lightness (V). These two components are measured for each pixel of the image and for both, we computed the mean and the standard deviation.

Here we summarize here the 4 variables measured to characterize lightness and saturation :

| LS_mean_satu | The mean saturation of an image. |
| --- | --- |
| LS_sd_satu | The standard deviation of LS_mean_satu. |
| LS_mean_light | The mean lightness of an image. |
| LS_sd_light | The standard deviation of LS_mean_light. |

## Body shapes (MO)

To measure the shape of the fish body outline we used morphometry statistics obtained with the *Momocs* *v1.3.2* R package [9]. This analysis relies on elliptical Fourier transformations: points are sampled along the fish body outline and transposed into a periodic function. This function is then decomposed into a sum of more simple functions like sine and cosine to re-transpose it to the object (Fig D.a). Each function represents an “harmonic” from low to high frequencies. Once the Fourier transformation was completed we computed a Principal Component Analysis (PCA) analysis on the elliptical Fourier transformations and used the first axes of the PCA to differentiate the fish silhouettes (Fig D.b). The Elliptical Fourier transformation function: *efourier* from the *Momocs v.1.3.2* R package requires defining the number of harmonics to use during the transformation. This value was chosen in preliminary analysis performed with the images available in Tribot et al. [7], where we found a higher correlation between the first axis of the PCA and fish aesthetic values with 15 harmonics compared with fewer or more harmonics.

Introduction to Momocs can be found here : https://momx.github.io/Momocs/articles/Momocs_intro.html

Here we summarize the 2 variables measured to characterize the shape of the fish body outline :

| MO_Fourier_pc1 | Position of the image on the first axis of the PCA. |
| --- | --- |
| MO_Fourier_pc2 | Position of the image on the second axis of the PCA. |

**B : IMAGE SAMPLING STRATEGY**

## Image database and copyrights

Most of the images gathered are under creative common licence but some have copyright and cannot be shared (although the numerical information contained in the image could be used to train our deep learning algorithm). Dataset A provides copyrights for the images used as illustrations in our figures. Dataset C provides the links to all original photographic material used in our study.

## Images used in the online survey

The images used as the learning set of the predictive model were a combination of the images already evaluated in Tribot et al. [7] study (157 images) and a set of 345 images used in a new survey (see main text) to get direct evaluation of species aesthetics. The new online survey shared 21 images with the Tribot et al. [7] study. The remaining 324 images were selected as follow:

1. We performed a PCA analysis using different image features on all the images of our collection (4,881 images). We used the following features (defined in Supplementary section 1): CL_cie_d_mean, CL_cie_d_sd, CL_hullarea, SH_n.core.cell_mean, SH_n.core.cell_sd, SH_perimeter_mean, SH_perimeter_sd, SH_perim.area.ratio_mean, SH_perim.area.ratio_sd, SH_core.area.index_mean, SH_core.area.index_sd, LS_mean_satu, LS_sd_satu, LS_mean_light, LS_sd_light, MO_Fourier_pc1 and MO_Fourier_pc2. The PCA was computed with the R *prcomp* function from the R package *stats v3.6.2*.
2. The five first dimensions of the PCA (representing 77% of the total explained variance) were used to construct a convex 5 dimensional volume. 162 images were randomly drawn near the vertices of this volume (from the vertice to 10% within the volume). The other 162 images were randomly drawn within the remaining volume.

We added to this pool of 324 images, 21 images with contrasted aesthetic values chosen from the 157 images previously evaluated in Tribot et al. [7]. This procedure allowed us to create a set of 345 images with representative features values of the whole set of images available (Fig E).

# C : SOCIO-CULTURAL BACKGROUND

*Questionnaire*

To ensure the robustness of the survey designed to evaluate the human preference for reef fishes, the photographic material had to be evaluated by a large panel of humans with contrasted socio-cultural background [4, 7]. To collect socio-cultural background in our survey, each participant was asked to answer a questionnaire (Fig F).

13,372 judges answered the online survey. The last question of the questionnaire was asked to detect judges with color vision issues (these judges were removed from the study; 2.78% of the judges). We thus ended up with a set of 13,000 judges' answers.

We found no difference in the probability for an image to win or not a match when the judge was under or over 14 years old (Kruskal-Wallis chi-squared = 0, p-value = 1), so we chose to keep only the questions that were answered by all judges: gender, age, education, experience with diving and spearfishing, fishkeeping, place of living, distance from the sea, exposure to natural space and knowledge about coral reef fishes. Fig G and Fig H provide an overview of the results obtained for the 13,000 judges.

*Socio-cultural background effect*

We tested the robustness of the matches’ outcome (probability for an image to win or not a match) to socio-cultural background. Using the *glmer* function from the *lme4 v 1.1-26* R package, we computed a generalized linear mixed model (GLMM) with a binomial error structure in which the image was considered as a random effect variable to order the socio-cultural variables according to their individual effect on the response variable. To reduce the number of combination tested by the model, we created age categories (<14; 15-25; 26-40; 41-60; >60 years) and country categories (France, United States of America, Europe, Others). Finally the socio cultural variables used were: gender (categorial) , age (ordered), education (ordered), scuba diving (categorial), country (categorial), experience with spearfishing (categorial), aquarium (categorial), place of living (ordered) and its distance from the sea (ordered), frequency of exposure to nature (ordered), knowledge about coral reef fishes (categorial). This analysis of variance for the first model (led with the function *Anova* from the *car v3.0-9* R package [10]) showed no effect of any of the socio-cultural variables (Table A) on the matches’ outcome. Thus, we did not conduct further analysis on the socio-cultural background of the judges and we used the answers of the 130,000 judges without color perception problem to compute the Elo scores.

# D : Elo SCORES

*Computation of elo scores*

Based on the answers of the 13,000 judges, scores were attributed to the 345 images included in the survey using the *Elo* algorithm [11] through the *EloChoice* *v0.29.4* R package [12] and with 1,000 randomizations of the order of the matches (Fig I). All species show rapid stabilization of their Elo score as the matches accumulate.

*Uniformization of the scores*

Among the 345 images, 21 had already been evaluated by Tribot et al. [13]. The r^2^ of the linear regression between the two scores of these 21 images was 0.89 (p-value < 0.001; Fig J). We used the intercept and slope of this relationship (Equation S1) to ‘correct’ the Elo score of the 157 images evaluated in Tribot et al. [7] and merge the two datasets.

$Elo\_Tribot\_corrected =109.1305 +(0.8955 \times Elo\_Tribot)$ *Equation S1*

**E : DEEP LEARNING ALGORITHM**

*Size of the images*

Deep learning algorithms are frequently trained with images of size 32×32, 64×64, 128×128, 224×224, 256×256 or 512×512 pixels [14]. The models we used, ResNet18 and ResNet50, were both pre-trained on 224×224 pixels ImageNet images [14, 15] whereas our images were standardized to 500×500 pixels. We thus tested if the size of the input images influenced the performances of the models and if so, which size produce the best performances. We trained both ResNet18 and ResNet50 (see main text for method) on the images of fishes resized at 32×32, 64×64, 128×128, 224×224, 256×256 or 512×512 pixels and compared the r^2^ of the linear regression between the predicted and evaluated scores of a testing set (Fig K). This procedure was repeated 5 times to avoid making conclusions on an unique run. ResNet50 performs better than ResNet18 for all sizes. ResNet50’s performances are equivalent for sizes of 224×224 and 512×512 pixels thus in order to save computation time we chose to use 224×224 pixels image size.

*Model and parameters*

For our final predictions we used a ResNet50 architecture that we slightly modified (Fig L) and parametrized to achieve our goal. To ensure that the predicted values were as close as possible to the estimated values, we used mean square error (MSE) as the loss function because it produces an important punitive effect when the prediction is too far from the expected value. We added a 0.5 dropout layer before the last fully connected layer. The batch size was set to 4. The training began with a learning rate of 1e-1 and we used a learning rate scheduler and an early stopping callback to avoid overfitting. We trained the last convolutional block and the regression layer for 50 epochs.

**F : RELATIONSHIP BETWEEN FEATURES AND AESTHETIC VALUES**

To estimate the individual contribution of each image feature (Supplementary section 1) we used a multiple regression approach. Multiple regressions were used to examine the variation in the predicted aesthetic values for the 4,881 images of our dataset explained jointly by all features and to rank the individual effect of each feature (17 variables, see Supplementary section 1: CL_cie_d_mean, CL_cie_d_sd, CL_hullarea, SH_n.core.cell_mean, SH_n.core.cell_sd, SH_perimeter_mean, SH_perimeter_sd, SH_perim.area.ratio_mean, SH_perim.area.ratio_sd, SH_core.area.index_mean, SH_core.area.index_sd, LS_mean_satu, LS_sd_satu, LS_mean_light, LS_sd_light, MO_Fourier_pc1, MO_Fourier_pc2).

We first computed a correlation matrix between all the features (using Pearson correlation coefficients): when two or more features were correlated (threshold r < 0.7), we kept only the feature with the highest correlation to the aesthetic value. 10 variables were finally selected: CL_cie_d_mean, LS_sd_light, LS_mean_satu, MO_Fourier_pc1, MO_Fourier_pc2, LS_mean_light, SH_perimeter_mean, SH_core.area.index_sd, SH_perim.area.ratio_sd, SH_n.core.cell_mean. We then created a linear model (with a Gaussian response) to explain aesthetic values. In this model, each feature was ordered according to its independent contribution to the total variation in the response variable. We eliminated non-significant terms using a backward selection procedure, to derive a minimal adequate model. The remaining 9 variables were : CL_cie_d_mean, LS_sd_light, LS_mean_satu, MO_Fourier_pc1, MO_Fourier_pc2, LS_mean_light, SH_perimeter_mean, SH_core.area.index_sd, SH_perim.area.ratio_sd. We found that the variation of the 9 selected variables explained a high proportion of the variation in the aesthetic values (r^2^ = 0.64, p < 0.001). The coefficients (scaled) of the final model were used to measure the contribution of each variable to the aesthetic value (Fig 2 from the main text).

**G : PHYLOGENETIC ANALYSIS**

*Evolutionary distinctiveness*

The Evolutionary Distinctiveness (ED) is defined as the distance in the evolutionary tree from a species to its nearest neighbor, which means it is high when the species is phylogenetically isolated i.e. has a long unshared branch in the phylogenetic tree [16]. For each species, ED was computed and averaged over the 100 randomly resolved trees (see main text Methods), and compared to species aesthetic values. We found a significant negative relationship indicating that phylogenetically isolated species tend to have low aesthetic values (Fig M).

*Phylogenetic signal*

We investigated a phylogenetic signal of the aesthetic value with Pagel's λ coefficient [17], which characterizes the relation between the similarity of a given trait (here the aesthetic value) and the phylogenetic distance between species. Pagel's λ represents the possibility to reconstruct the tree with the studied trait only. A null λ would lead to a single polytomy for the basal node while a value of 1 would give the exact tree [18, 19]. We also computed Pagel's λ in each family in order to identify those for which an inner signal is detected. Table B provides Pagel's λ for the 57 families in our dataset for which we have five species or more and the coefficient for the global tree. Each λ is shown with the associated p-value of the likelihood ratio-test. Fig N provides mean aesthetic values of families with more than 10 species presented in decreasing order.

**H : ECOLOGICAL TRAITS**

We extracted eight ecological traits (Table C) from the RLS trait database [20]. Among the 2,417 species of our dataset, the eight traits were all available for 2,181 species, incomplete at less than 50% for 107 species and missing or incomplete at more than 50% for 129 species. To work with the largest possible species list, we used a *missForest* algorithm (R package *missForest* *v1.4* [21]) to predict the traits of the 107 species with less than 50% missing traits. Our dataset had 0.9 % of missing values yet the *missForest* was tested on a testing dataset where we artificially created 5 % of missing values. On this artificial dataset the accuracy of the predictions of the *missForest* for each trait were between 98% and 99.99%. This high accuracy allowed us to use the *missForest* algorithm to predict the missing values of the 107 species with less than 50% missing traits. The ecological traits analyses were thus finally led on 2,288 species (94.7% of the dataset). Aesthetic values were also compared among categories or along values of each RLS trait (Fig O).

**I : MEAN AESTHETIC VALUE**

Here, we show that our main findings are robust if the aesthetic value is computed by using the mean value among all images available for each species rather than the maximum value (as used in our main results). Given that for most species we have one (949 species) or two (871) images (mean number of images per species is 2.02; Fig P), we found a very high correlation between the mean and the maximum aesthetic values (Fig Q). To test the robustness of our main findings, we reproduced our main analyses with the mean aesthetic value. We found the same negative relationship between the mean aesthetic value and respectively the age of the species and their ecological distinctiveness (Fig R.a and Fig R.b). Pagel's λ, estimated on the entire tree, confirms that the phylogenetic signal is still strong when using the mean aesthetic value (λ = 0.74 ± 0.01, p-value < 0.001). We also observed the same tendencies for the IUCN status and fishery importance (Fig S).


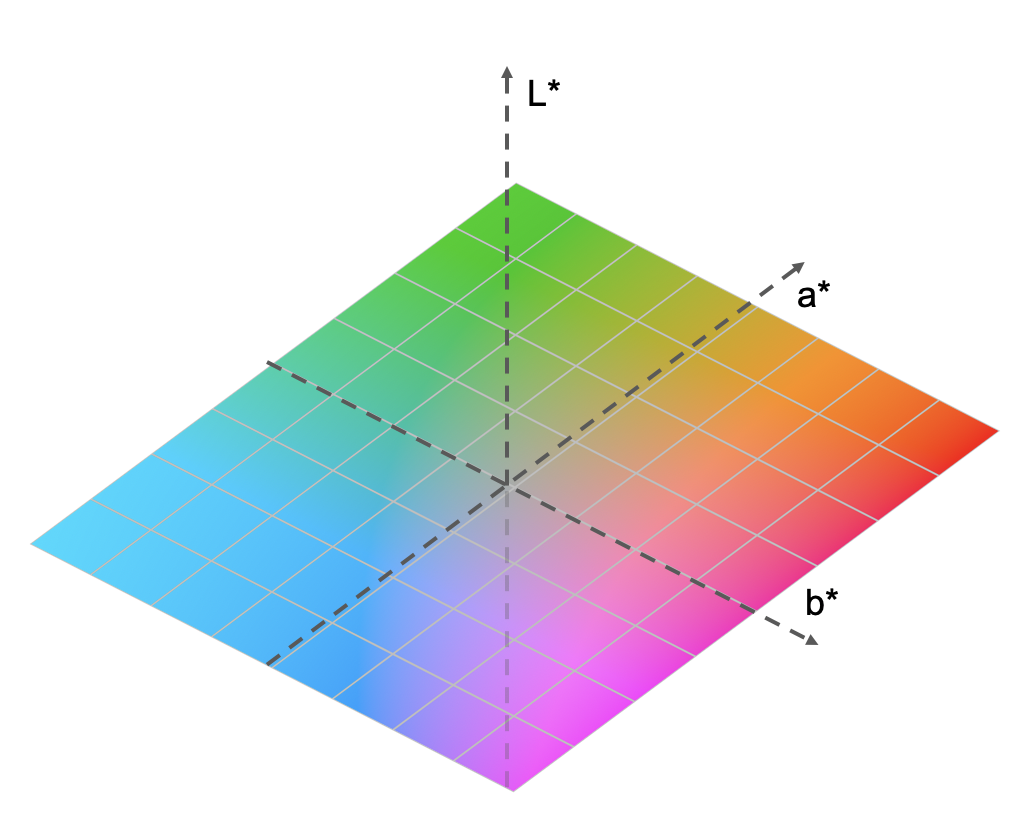


S1 Fig. A. Representation of the three dimension CIELAB space : *L** for lightness, and *a** and *b** for the four unique colors of human vision: red, green, blue, and yellow. Data and code required to generate this Figure can be found in https://github.com/nmouquet/RLS_AESTHE.


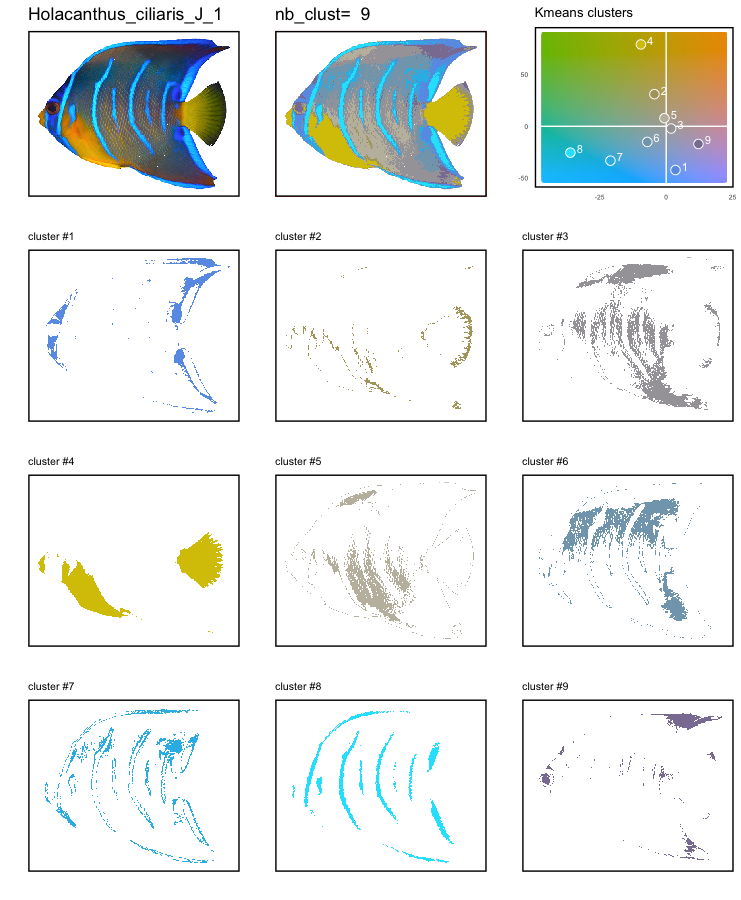


**S1 Fig. B. Cluster analysis performed for *Holacanthus ciliaris*.** Top left panel : original image. Top middle panel: image created with the 9 clusters and corresponding colors. Top right panel: projection of the 9 clusters centroids on the a*b* color space. The 9 other panels show the pixels belonging to each color cluster. See Dataset A for copyrights. Data and code required to generate this Figure can be found in https://github.com/nmouquet/RLS_AESTHE.


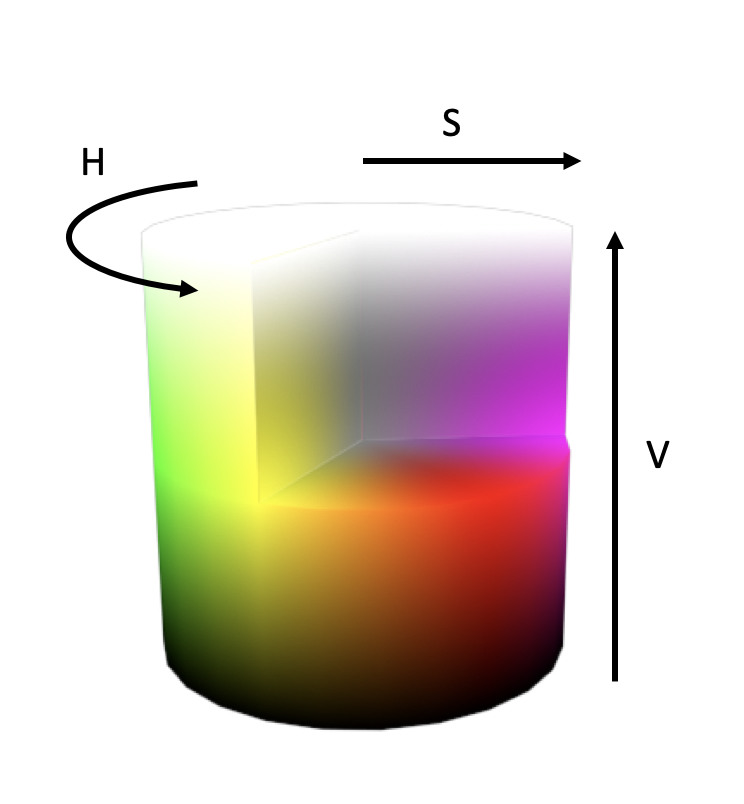


**S1 Fig. C. HSV color space.** Modified from:

<https://fr.wikipedia.org/wiki/Teinte_saturation_lumi%C3%A8re#/media/Fichier:Cylindre.png>


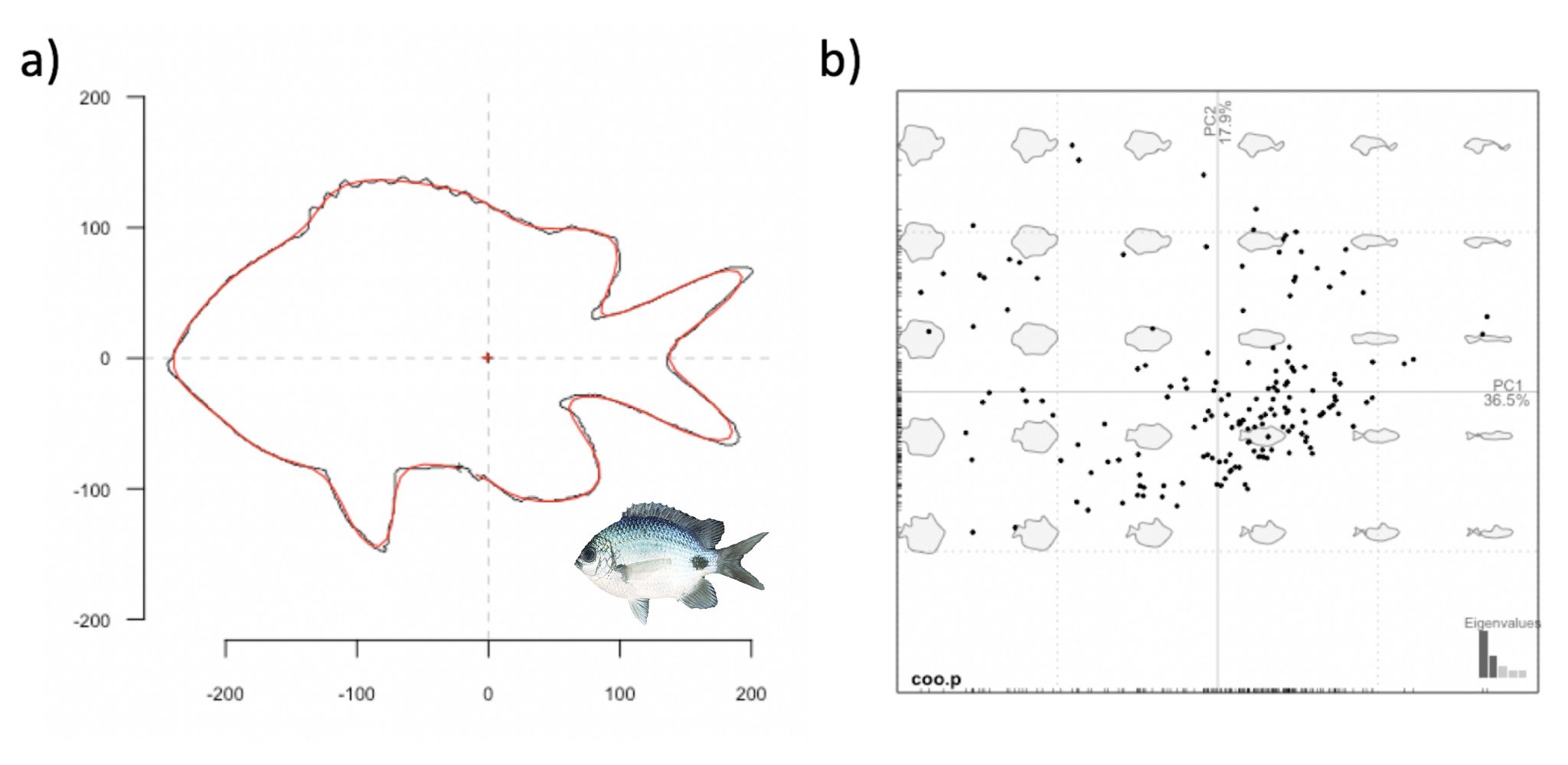


**S1 Fig. D. Illustration of the analysis led with the Momocs package.** (a) Elliptical Fourier transformation on *Abudefduf sparoides* with 15 harmonics. The black line corresponds to the original fish body outline and the red line to the elliptical fourier transformation. (b) Projection of the 157 fish from Tribot et al. [7] on the two first axes of the PCA analysis based on the elliptical Fourier transformation. The first axis of the PCA explains most of the variation and clearly differentiates circular from elongated fishes. See Dataset A for copyrights. Data and code required to generate this Figure can be found in https://github.com/nmouquet/RLS_AESTHE.


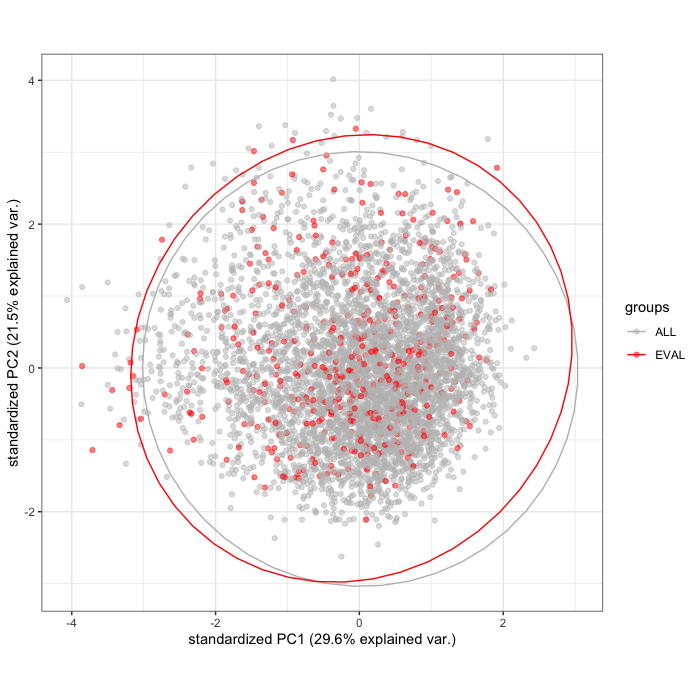


**S1 Fig. E.** Projection of the 4,881 images of our collection (“ALL”) and of the 345 images for which we obtained direct evaluation of aesthetic values from the online survey (“EVAL”), on the two first axes of the PCA on the images’ features. The two ellipses show the 99% confidence intervals. This illustrates that the set of images used as the learning set (“EVAL”) is representative of the whole set of images available in our collection. Data and code required to generate this Figure can be found in https://github.com/nmouquet/RLS_AESTHE.


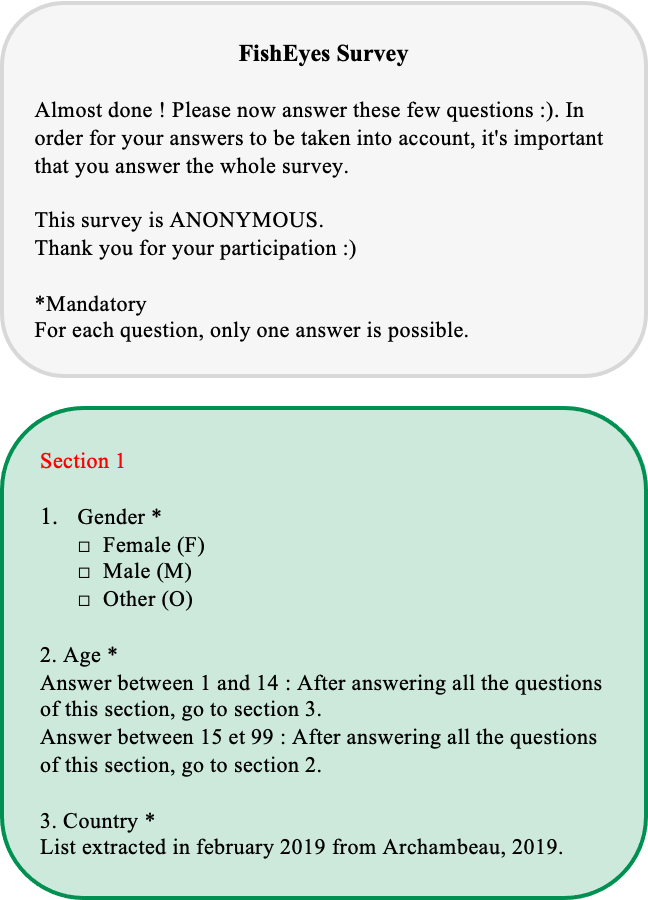


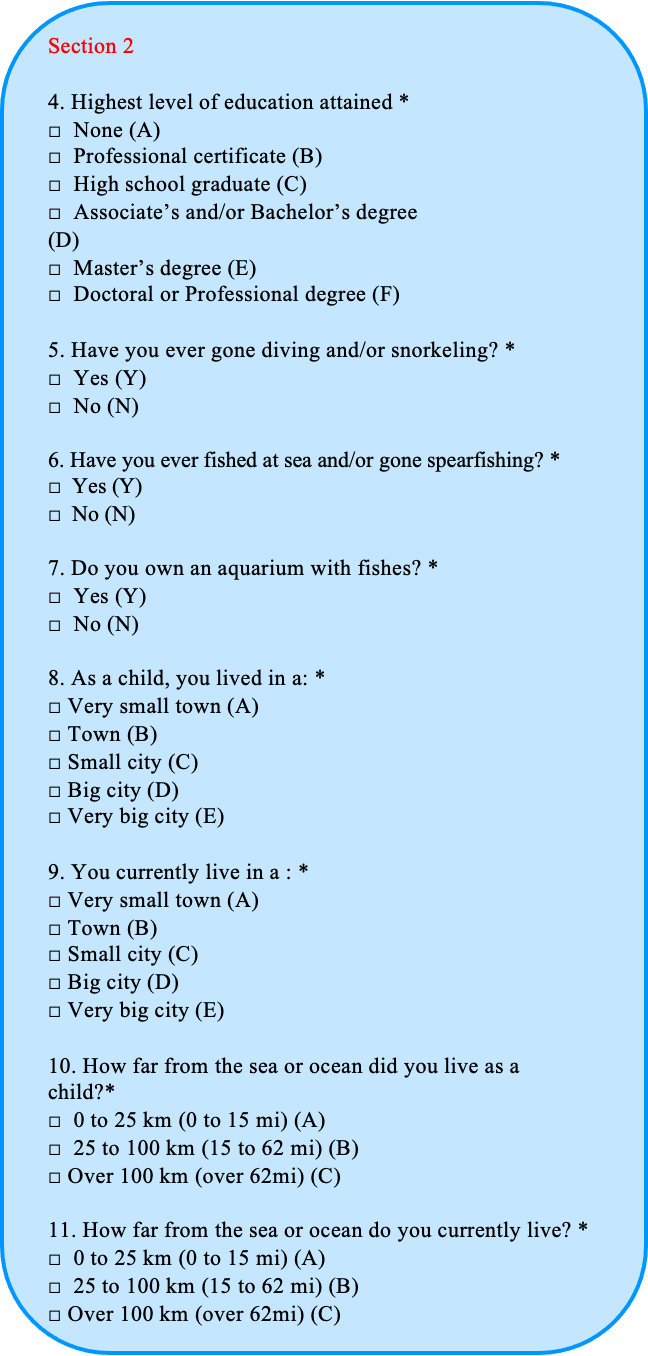


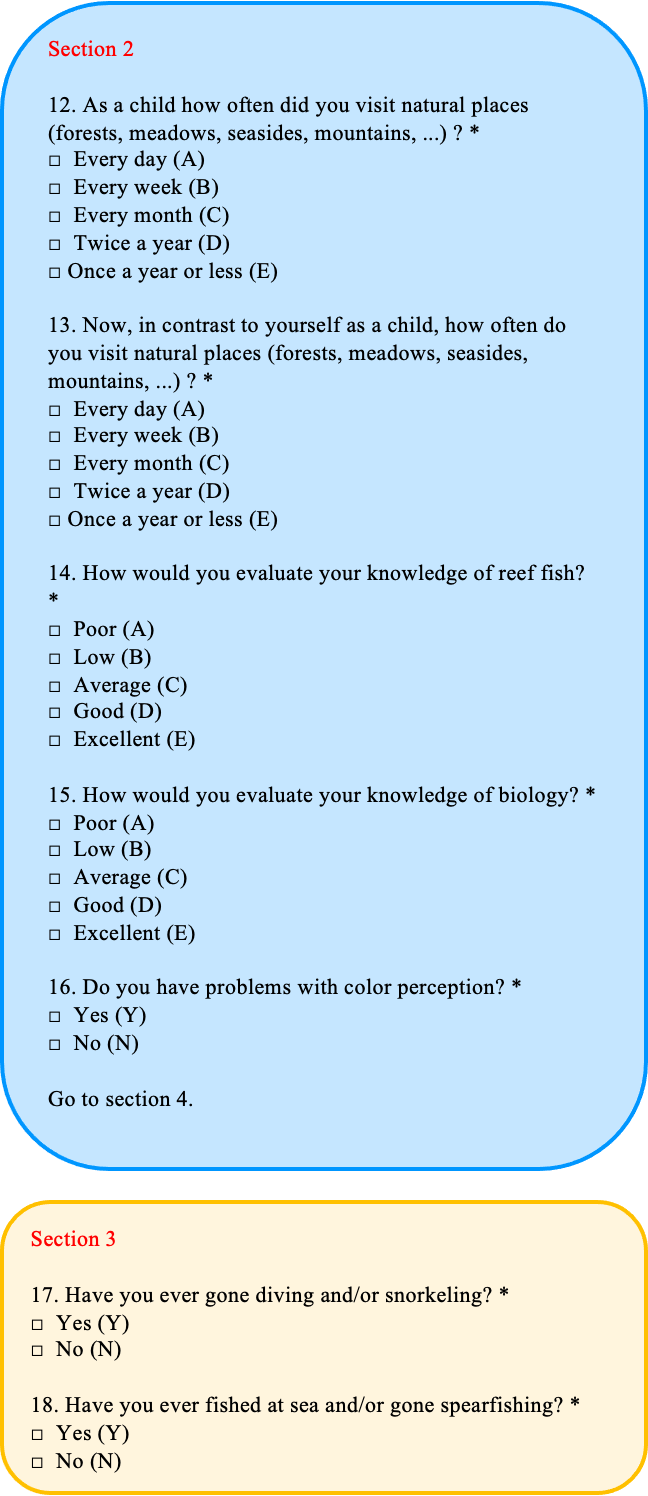


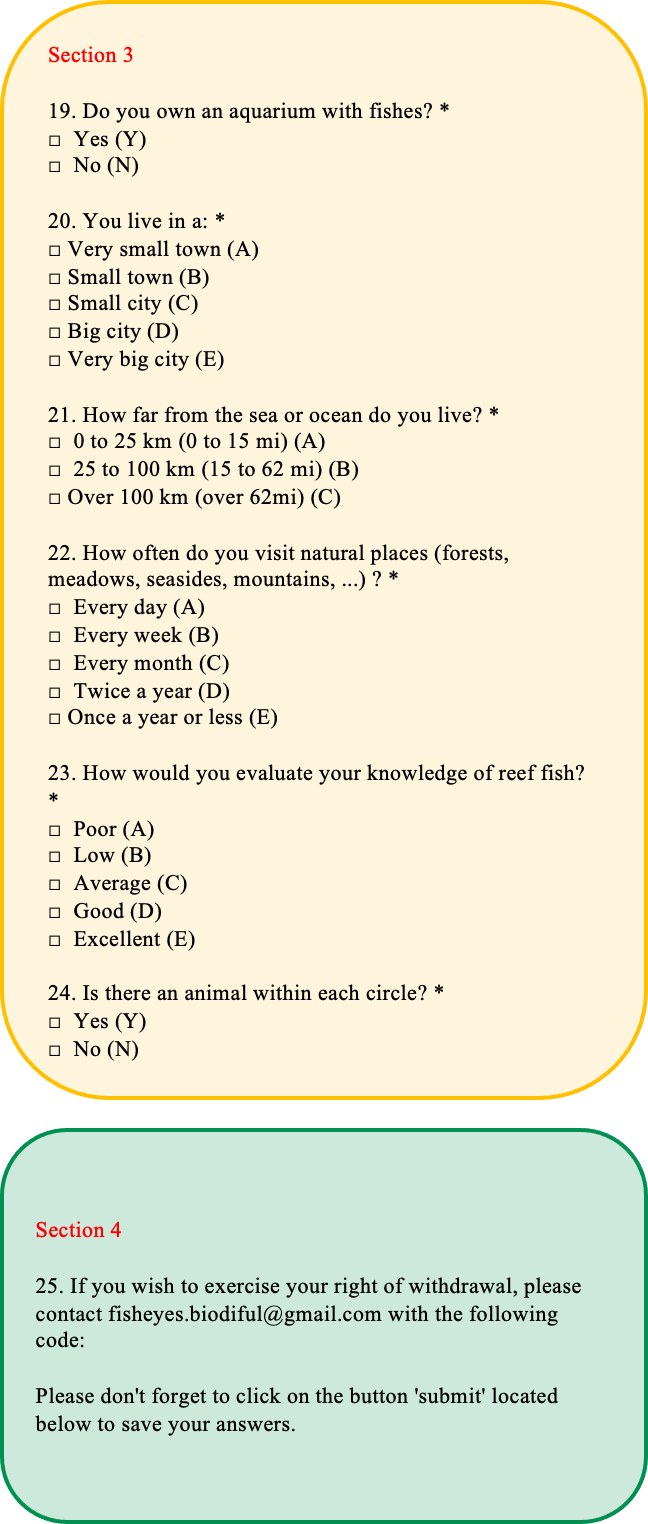

**S1 Fig. F. Questionnaire on the socio-cultural background of the judges.** Questions in green boxes were answered by all judges, questions in blue boxes were answered by judges over 14 years old and questions in yellow boxes were answered by judges under 14 years old.


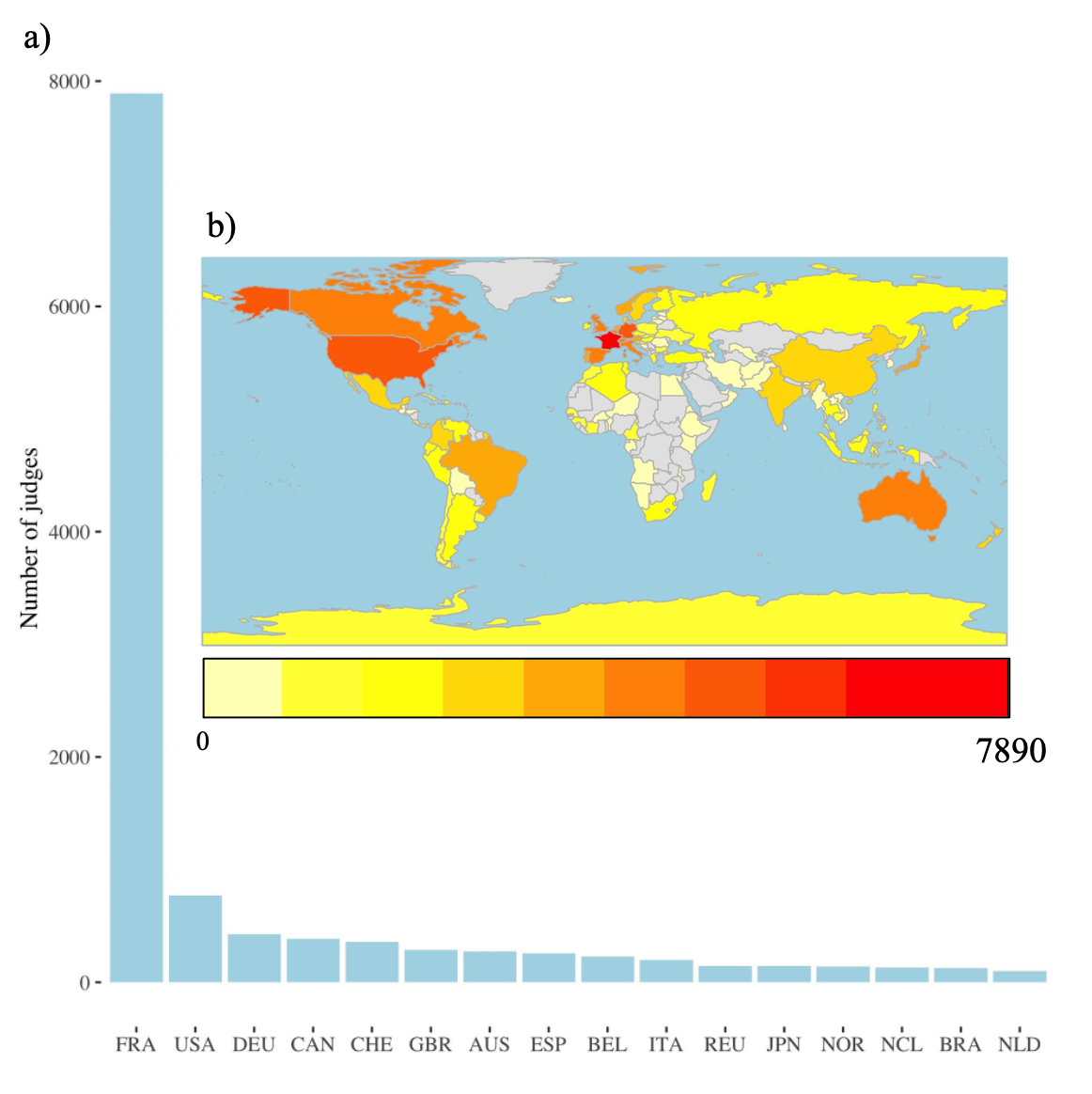


**S1 Fig. G. Number of judges per country**. **a)** Barplot of the number of judges for the ten countries with the highest number of judges. The codes used to designate the countries are the ISO 3166 country codes. **b)** Map of the number of judges per country. Countries with no judges are in gray. The number of judges was log transformed to create the color gradient. Data and code required to generate this Figure can be found in <https://github.com/nmouquet/RLS_AESTHE>. The map was obtained from rworldmap v1.3.6 R package which uses Natural Earth as base layer (<https://www.naturalearthdata.com/downloads/10m-physical-vectors/>).


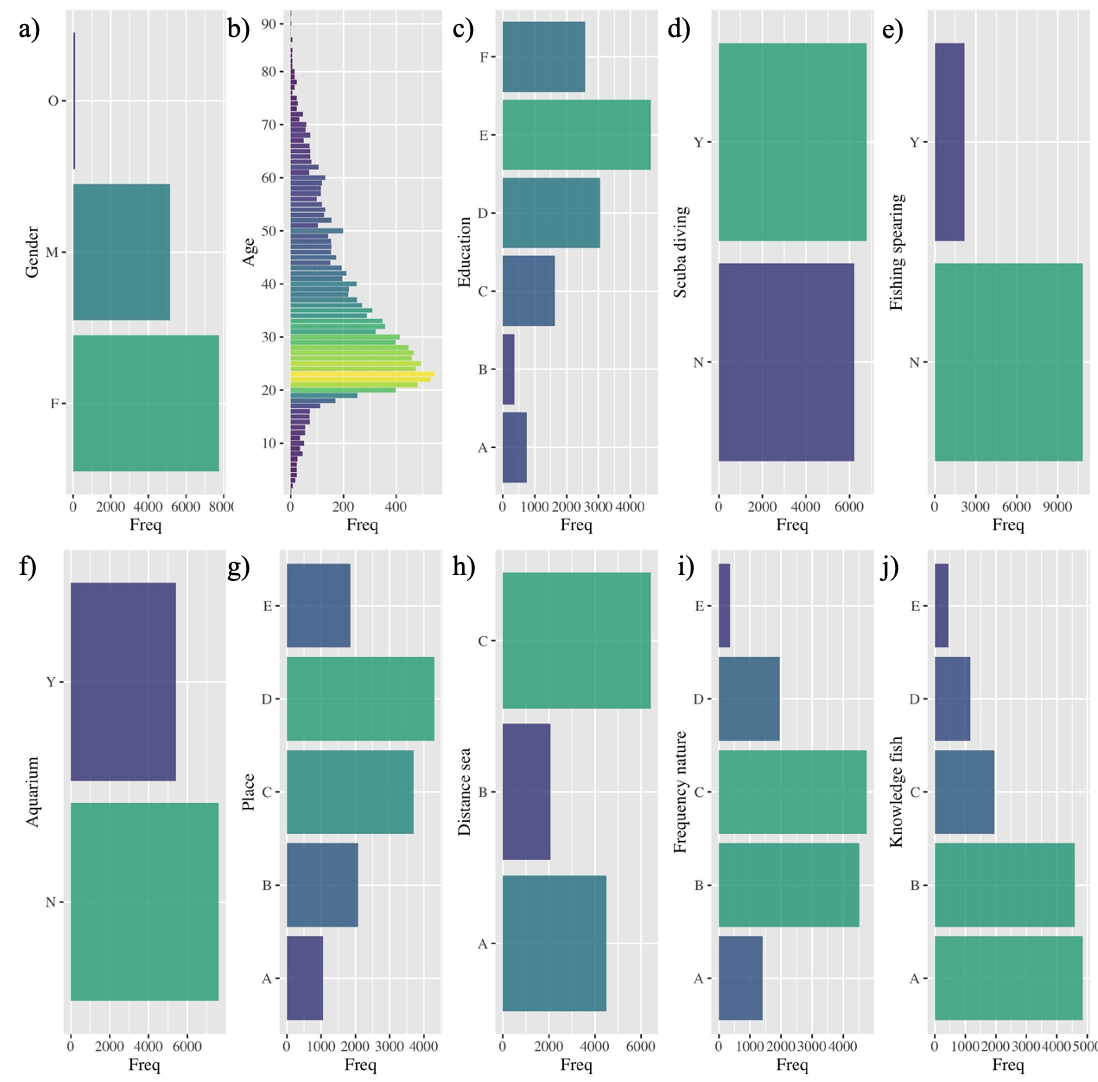


**S1 Fig. H. Summary of socio-cultural background of the 13,000 judges**. For the 10 panels, the bars are colored according to the frequency of the answer. **a)** gender, **b)** age, **c)** education, **d)** experience with diving, **e)** experience with spearfishing, **f)** fishkeeping, **g)** place of living and its **h)** distance from the sea, **i)** frequency of exposure to natural places, **j)** knowledge about coral reef fishes (see Fig F for modalities). Data and code required to generate this Figure can be found in https://github.com/nmouquet/RLS_AESTHE.


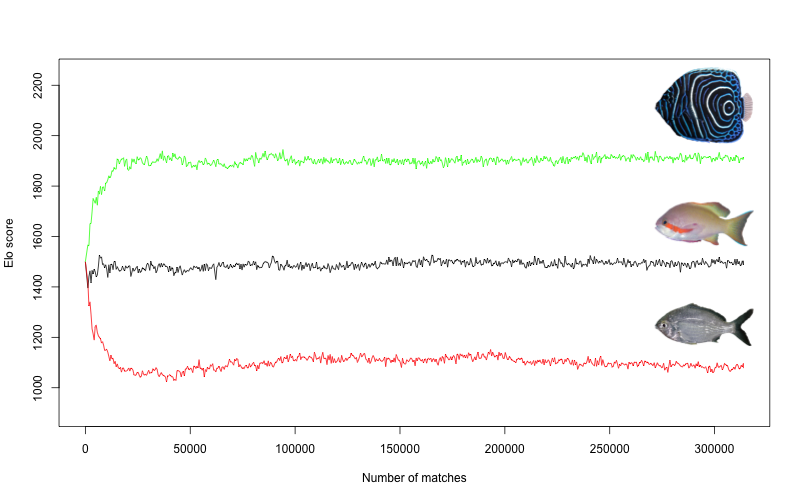


**S1 Fig. I. Variation of the Elo scores of the images as matches accumulate** (means from the 1,000 bootstrapings) for *Pomacanthus imperator* (green line), *Pseudanthias huchtii* (black line) and *Gerres subfasciatus* (red line). See Dataset A for copyrights. Data and code required to generate this Figure can be found in https://github.com/nmouquet/RLS_AESTHE.


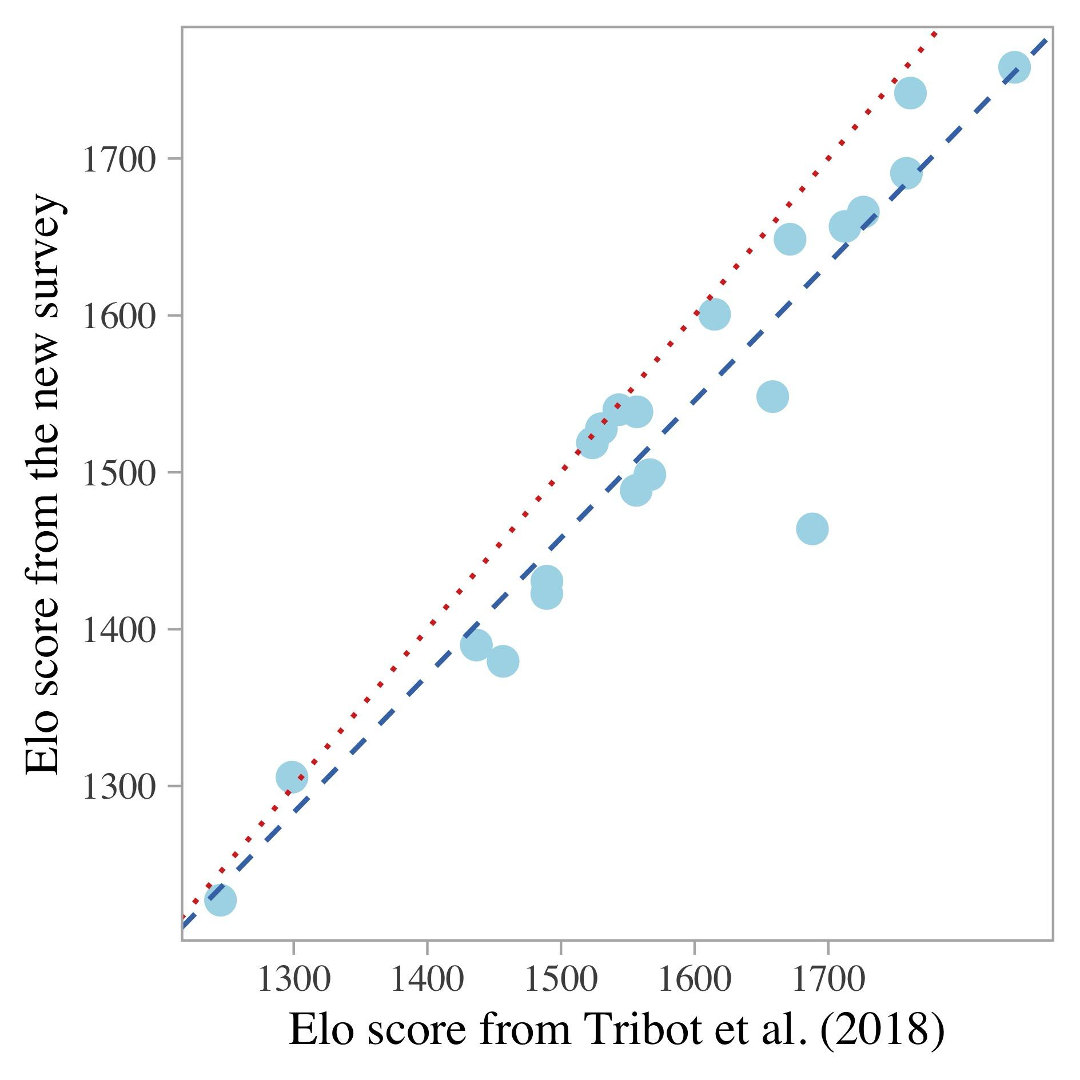


**S1 Fig. J.** Linear regression between the Elo scores computed in Tribot et al. [7] and in the new survey for the 21 images in common between the two surveys (r^2^ = 0.89, p-value < 0.001). The red dotted line shows the 1:1 relationship. Data and code required to generate this Figure can be found in https://github.com/nmouquet/RLS_AESTHE.


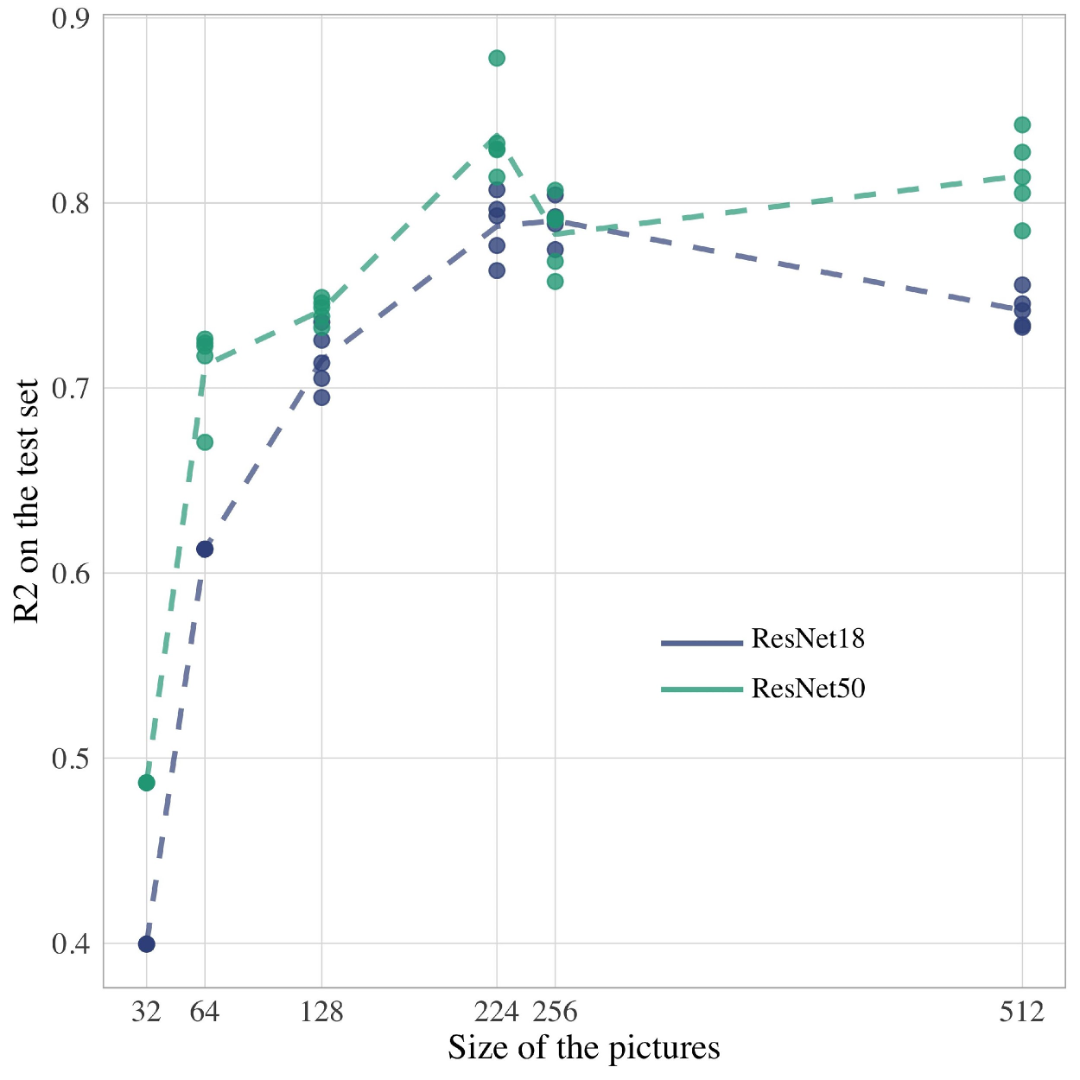


**S1 Fig. K. Effect of the size of the input images on the performances of the model.** Each point represents the r^2^ coefficient of the linear relationship between the evaluated scores and the scores predicted by either ResNet18 (in blue) or ResNet50 (in green) trained on images of a given size. The dotted lines show the average performances of the ResNet18 (in blue) and the ResNet50 (in green). Data and code required to generate this Figure can be found in https://github.com/nmouquet/RLS_AESTHE.


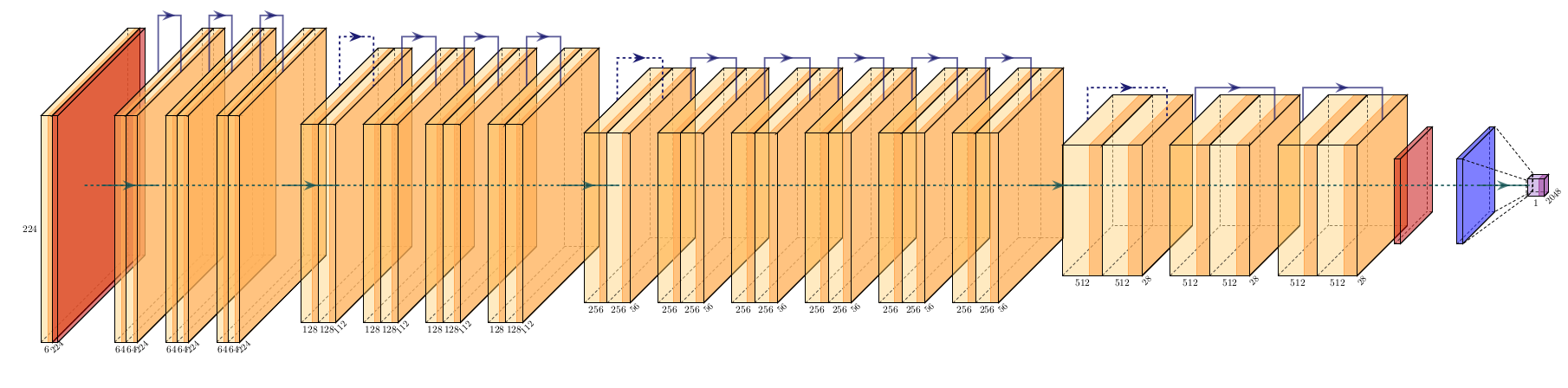


**S1 Fig. L. Architecture of ResNet50 modified to predict the aesthetic values.** The first layer (in pale orange) is a convolutional layer and the second layer (in dark orange) a pooling layer. The model includes four convolutional blocks respectively made of three, four, six and three convolutional layers. A last pooling layer (in dark orange) is linked to a dropout layer (in blue). The last layer is a fully connected layer (in purple) that outputs a continuous aesthetic value. Illustration inspired from the PlotNeuralNet [22] Python library. Data and code required to generate this Figure can be found in https://github.com/nmouquet/RLS_AESTHE.


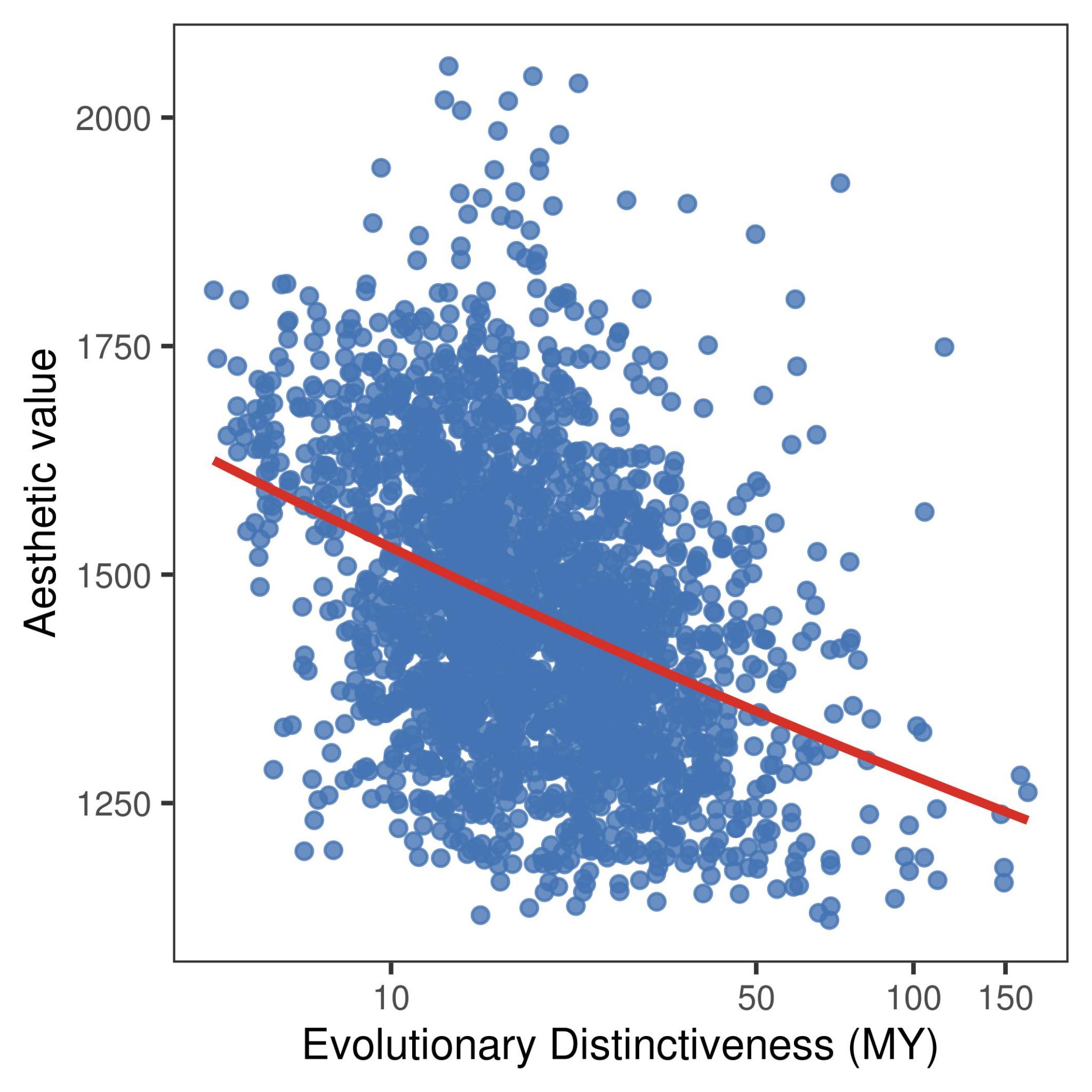


**S1 Fig. M. Relationship between the Evolutionary Distinctiveness of species (in MY) and their aesthetic value**. With ED log transformed, the relationship is significantly negative (r^2^ = 0.14, p-value < 0.001): Species with high aesthetic values tend to be less isolated in the evolutionary tree than species with lower aesthetic values. Data and code required to generate this Figure can be found in https://github.com/nmouquet/RLS_AESTHE.

##
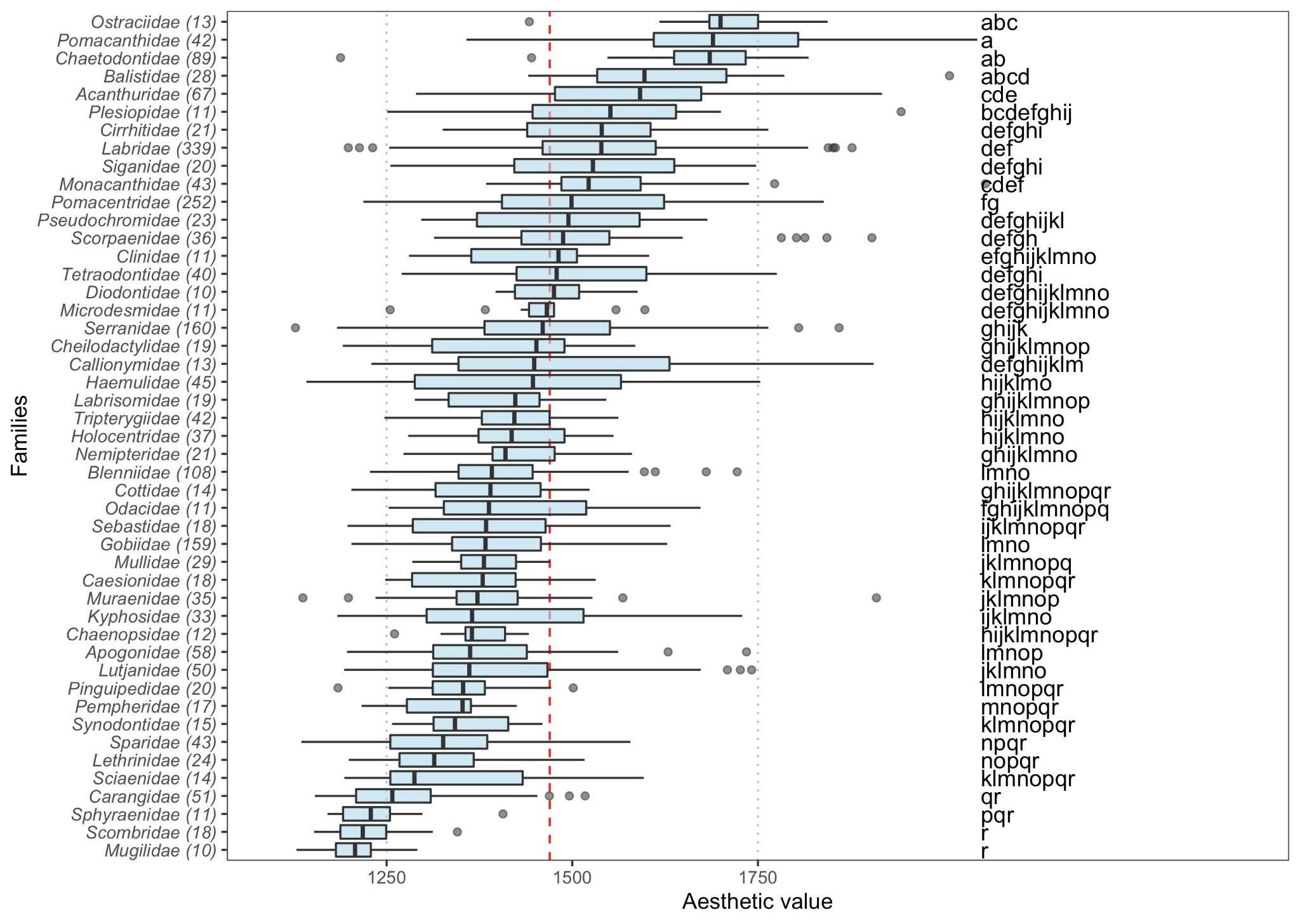


**S1 Fig. N. Mean** **aesthetic values of families with more than 10 species presented in decreasing order.** The numbers in brackets refers to the number of species of the family in our dataset. A one-way ANOVA test show a strong effect of families (p-value < 0.001); The letters on the right indicates families belonging to similar groups (Tukey’s post-hoc tests, p-value < 0.05; for all p-values of the Tukeys’s tests, see the code analysis/01_phylogeny.R in the GitHub Repository: https://github.com/nmouquet/RLS_AESTHE). The red dashed line shows the mean aesthetic value of all species. Data and code required to generate this Figure can be found in https://github.com/nmouquet/RLS_AESTHE.


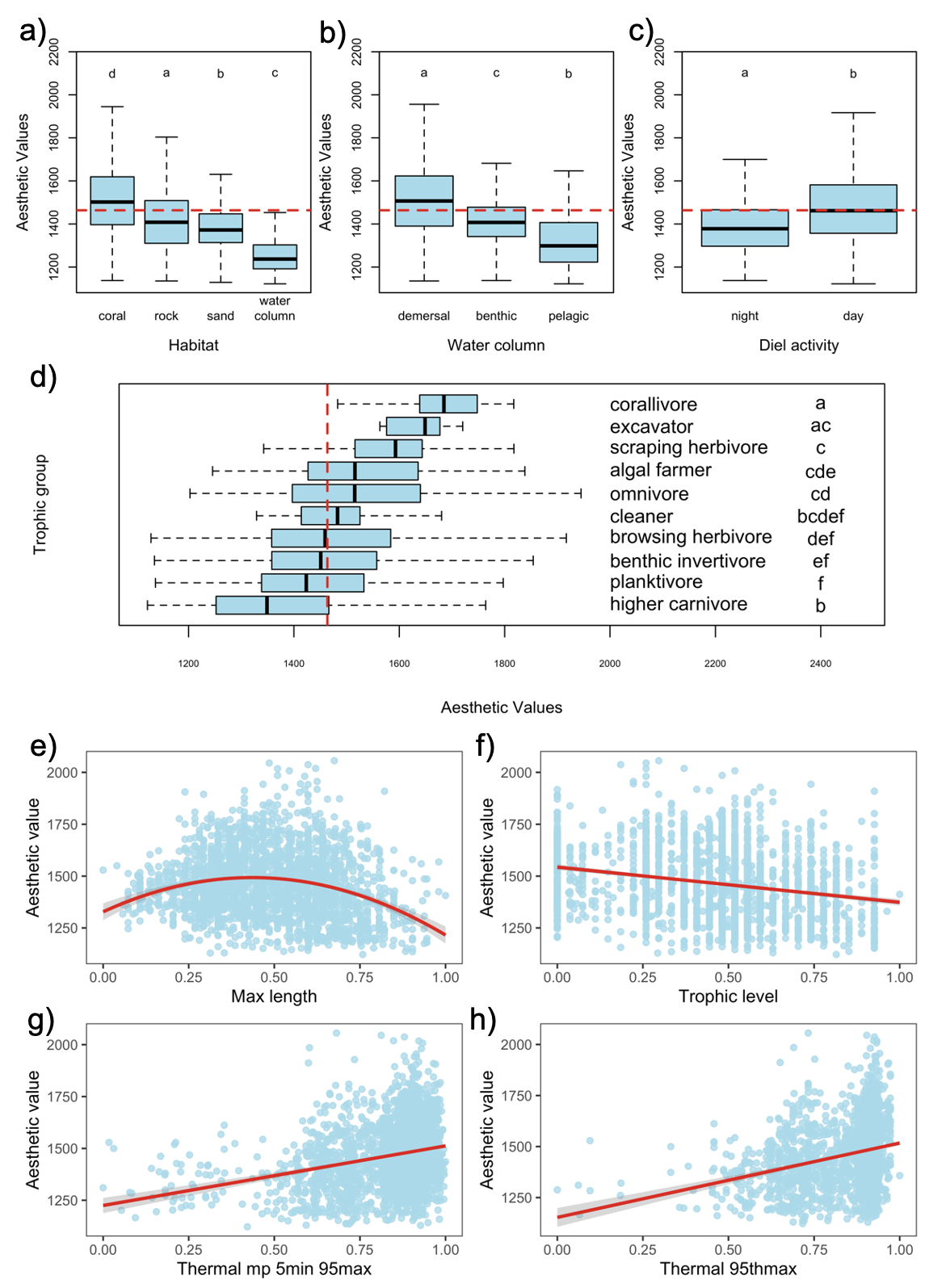


**S1 Fig. O. Comparison between the aesthetic value of the fish species and their ecological traits** (File S1 Table C**)**. On the boxplots, the red dashed lines show the mean aesthetic value over all species and the letters indicate significant different distribution between groups (p-value < 0.05) via one-way ANOVA and Tukey’s post-hoc tests. Modalities who share common letters are not significantly different (See Table D for all p-values of the Tukey’s tests). **a)** Variation of the aesthetic value of fishes with their habitat (one-way ANOVA p value < 0.001). **b)** Variation of the aesthetic value of fishes with their position in the water column (one-way ANOVA p value < 0.001). **c)** Variation of the aesthetic value of fishes with their diel activity (one-way ANOVA p value < 0.001). **d)** Variation of the aesthetic value of fishes with their trophic group (one-way ANOVA p value < 0.001). **e)** Polynomial relationship between the aesthetic value of species and their maximum length log transformed and normalized (r^2^ = 0.058; p-value < 0.001). **f)** Linear relationship between the aesthetic value of species and their trophic level log transformed and normalized (r^2^ = 0.06; p-value < 0.001). **g)** Linear relationship between the aesthetic value of species and the 5th quantile in their temperature distribution log transformed and normalized (r^2^ = 0.069; p-value < 0.001). **h)** Linear relationship between the aesthetic value of species and the 95th quantile in their temperature distribution log transformed and normalized (r^2^ = 0.073; p-value < 0.001). Data and code required to generate this Figure can be found in https://github.com/nmouquet/RLS_AESTHE.

**
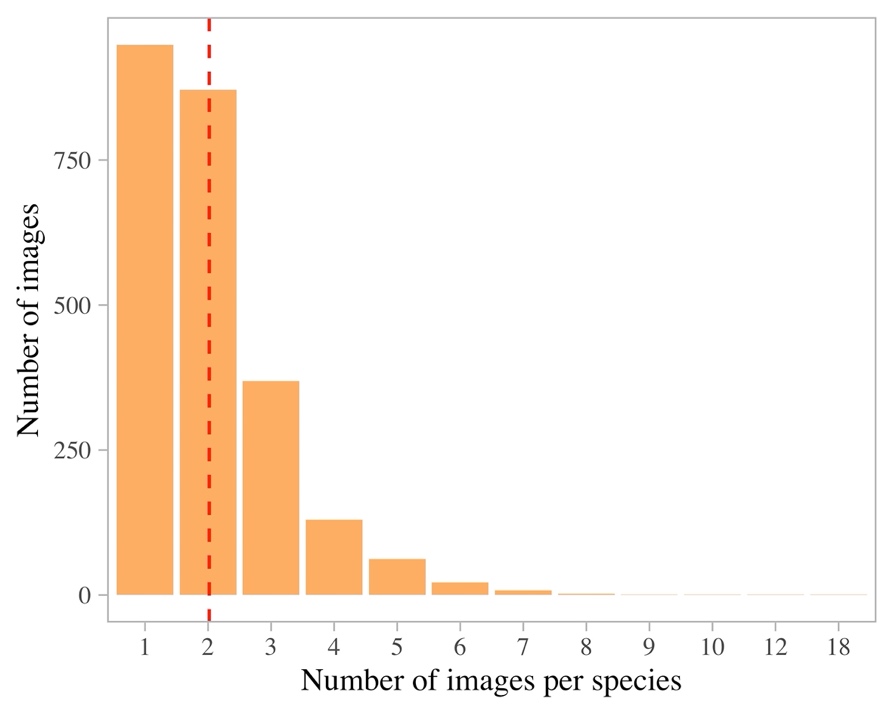
**

**S1 Fig. P. Number of images per species.** This histogram shows the number of images available per species among our 4,881 images. The red dashed line shows the mean number of images per species in the entire database. Data and code required to generate this Figure can be found in https://github.com/nmouquet/RLS_AESTHE.


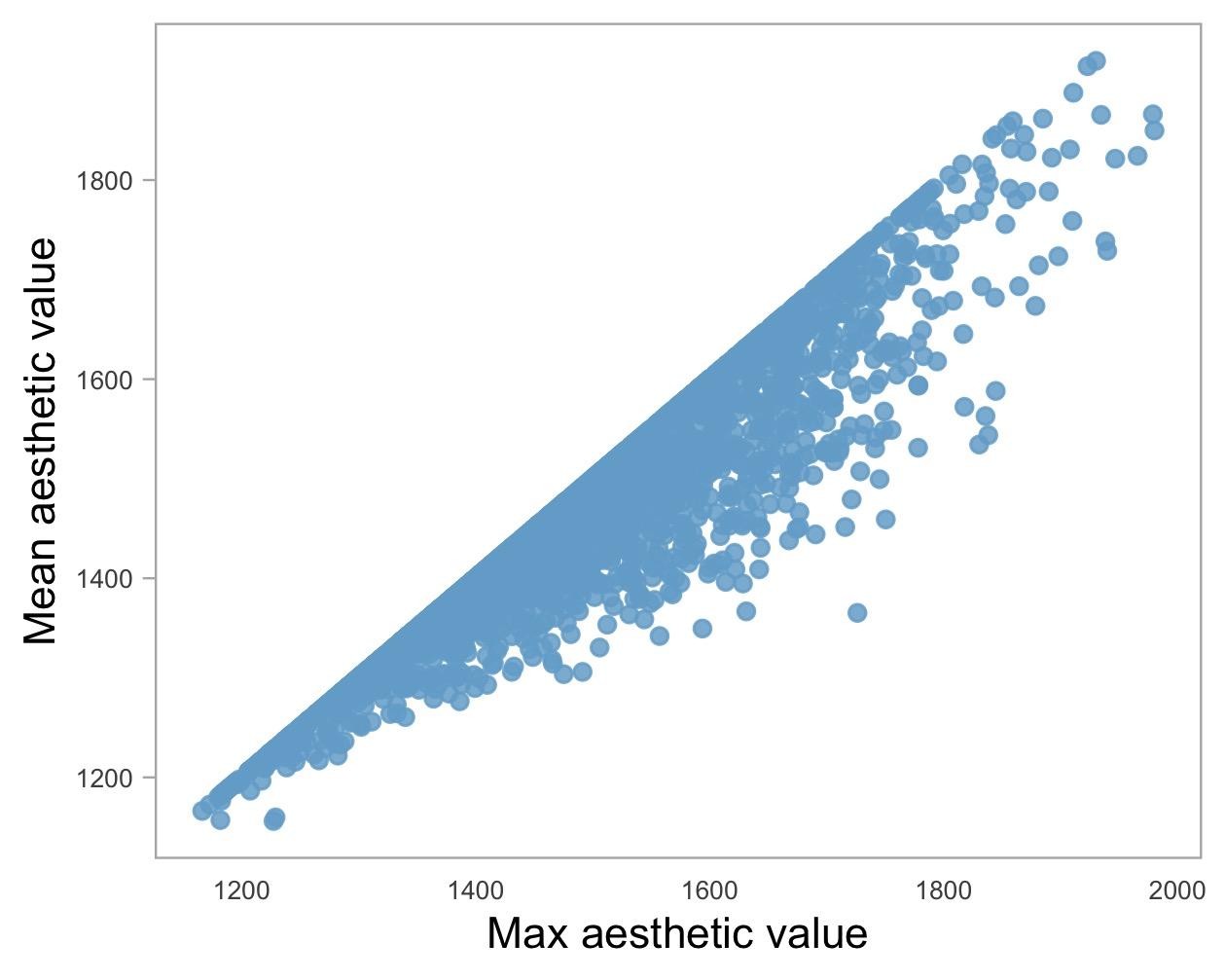


**S1 Fig. Q.** Relationship between the aesthetic values computed using the maximum value for each species (as used in our main results) and the aesthetic values computed using the mean value among all images available for each species (r^2^ of the linear relationship = 0.89, p-value < 0.001). Data and code required to generate this Figure can be found in https://github.com/nmouquet/RLS_AESTHE.


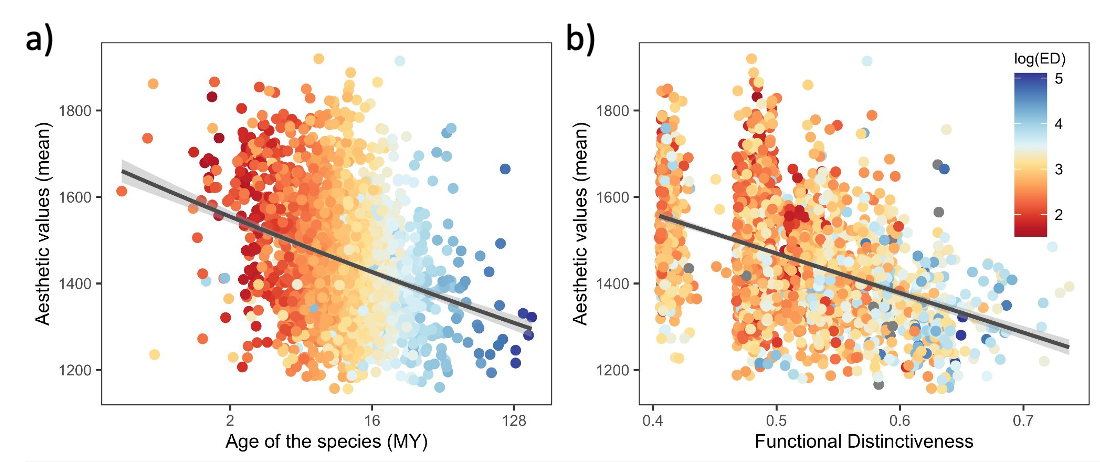


**S1 Fig. R. Phylogenetic history and ecological originality with mean aesthetic values.** **a)** Relationship between the mean aesthetic values of species and their age in Millions of years (averaged over the 100 random trees) (r^2^ = 0.10, p-value < 0.001). **b)** Relationship between the mean aesthetic values and their Ecological Distinctiveness (r^2^ = 0.19, p-value < 0.001). On both panels, species Evolutionary Distinctiveness (averaged over the 100 random trees and log transformed) have been used to color the points from low (dark red) to high (dark blue) values. Data and code required to generate this Figure can be found in https://github.com/nmouquet/RLS_AESTHE.


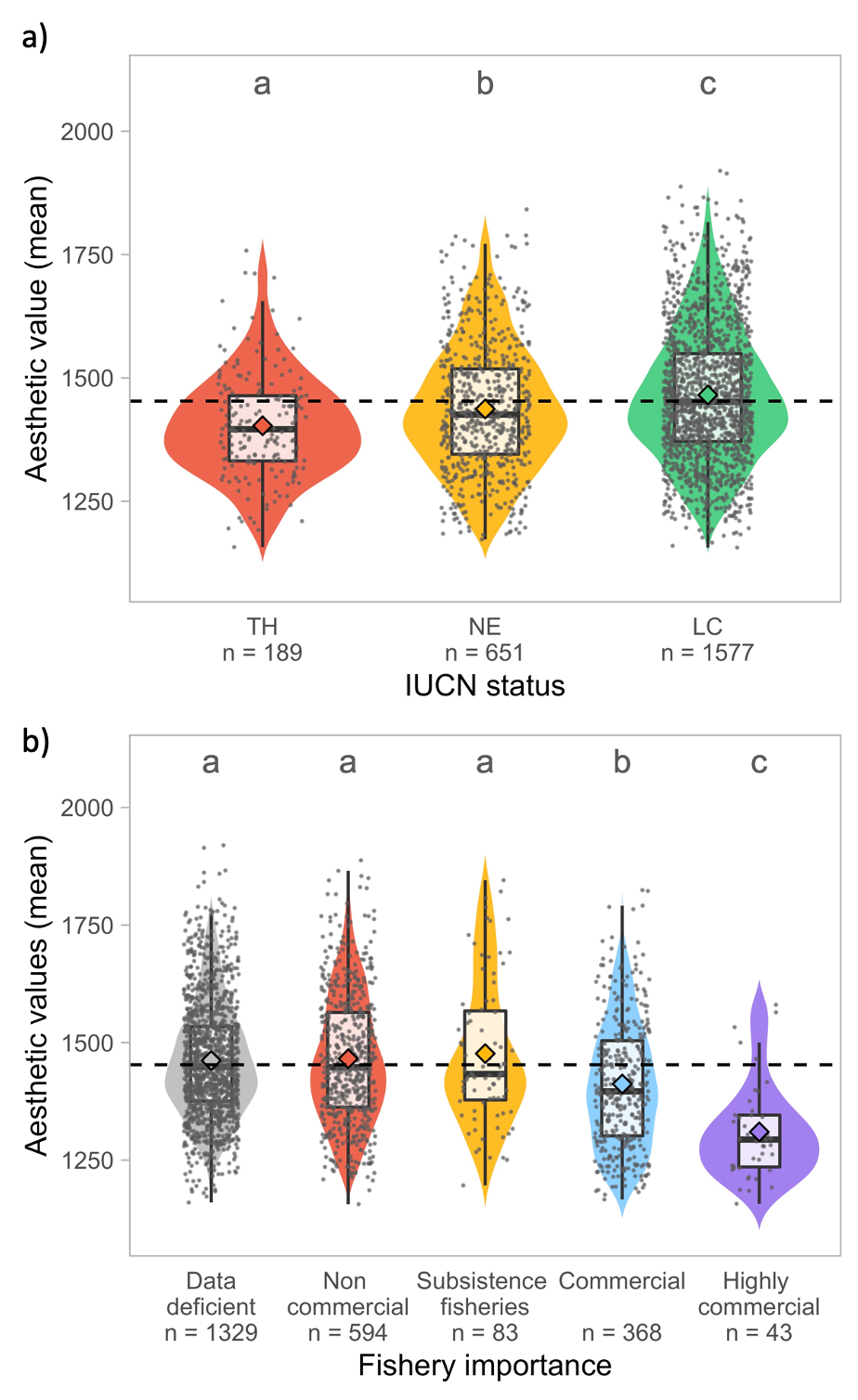


**S1 Fig. S. Conservation status.** **a)**Violin plot of the mean aesthetic values of reef fishes for three groups of conservation status: Threatened (TH), Not Evaluated (NE) and Least Concerned (LC). Letters indicate significant differences between the groups (Tukey p-values are respectively p < 0.01 between TH and NE, p < 0.001 between LC and TH and p < 0.001 between LC and NE). **b)**Violin plot of the aesthetic values of reef fishes for the five groups of fishery importance : “Data deficient” = no data available; “Non commercial” = no interest for fisheries or potential interest or minor interest ; “Subsistence fisheries” = importance for subsistence fisheries ; “Commercial” = commercial importance for fisheries ; “Highly commercial” = high commercial importance for fisheries. Letters indicate significant differences between the groups (all Tukey p-values are < 0.001). Data and code required to generate this Figure can be found in https://github.com/nmouquet/RLS_AESTHE.

**S1 Table A.** **Analysis of Deviance in the generalized linear mixed model.** The p-values of the chi-square tests are > 0.5 indicating that none of the considered variables have a significant effect on the probability for an image to win a match. Data and code required to generate this Table can be found in https://github.com/nmouquet/RLS_AESTHE.

| Variable | Chisq | Df | p-value |
| --- | --- | --- | --- |
| Gender | 0.011 | 2 | 0.994 |
| Age | 0.635 | 4 | 0.959 |
| Education | 0.405 | 5 | 0.995 |
| Scuba diving | 0.223 | 1 | 0.637 |
| Country | 0.218 | 3 | 0.975 |
| Spearfishing | 0.099 | 1 | 0.753 |
| Aquarium | 0.036 | 1 | 0.849 |
| Place of living | 0.928 | 4 | 0.920 |
| Distance from the sea | 0.058 | 2 | 0.971 |
| Frequency of exposure to nature | 0.548 | 4 | 0.969 |
| Knowledge about coral reef fishes | 0.676 | 4 | 0.954 |

**S1 Table B. Pagel’s λ.** The coefficient of the whole tree is 0.74 ± 0.01 (p-value < 0.001). The other highlighted lines indicate the families for which λ is higher than 0.5 and the p-value lower than 0.05. The last two columns of the table are the number of species in our dataset for each family and the percentage it represents compared to the total number of species in each family according to the WoRMS database [23, 24]. Data and code required to generate this Table can be found in https://github.com/nmouquet/RLS_AESTHE.

| Family | Pagel's lambda | Standard error | p-value | Number of species | % |
| --- | --- | --- | --- | --- | --- |
| Acanthuridae | 0.707 | 0.094 | 0.001 | 67 | 55.4 |
| Apogonidae | 0.161 | 0.263 | 0.856 | 49 | 9.5 |
| Balistidae | 0.842 | 0.000 | 0.006 | 28 | 19.7 |
| Batrachoididae | 0.149 | 0.220 | 0.805 | 9 | 5.1 |
| Blenniidae | 0.107 | 0.064 | 0.336 | 106 | 18.7 |
| Caesionidae | 0.097 | 0.244 | 0.988 | 18 | 25.4 |
| Callionymidae | 0.071 | 0.226 | 0.952 | 13 | 5.3 |
| Carangidae | 0.607 | 0.092 | 0.043 | 49 | 17.1 |
| Chaenopsidae | 0.047 | 0.207 | 0.974 | 11 | 7.5 |
| Chaetodontidae | 0.610 | 0.047 | 0.000 | 89 | 46.8 |
| Cheilodactylidae | 0.402 | 0.325 | 0.752 | 19 | 25.0 |
| Cirrhitidae | 0.448 | 0.304 | 0.458 | 21 | 23.3 |
| Clinidae | 0.090 | 0.236 | 0.912 | 11 | 5.9 |
| Clupeidae | 0.000 | 0.000 | 1.000 | 7 | 2.1 |
| Cottidae | 0.715 | 0.393 | 0.459 | 14 | 6.3 |
| Diodontidae | 0.010 | 0.059 | 0.981 | 10 | 11.4 |
| Embiotocidae | 1.000 | 0.000 | 0.268 | 9 | 12.0 |
| Ephippidae | 0.367 | 0.223 | 0.610 | 8 | 21.6 |
| Gadidae | 0.000 | 0.000 | 1.000 | 6 | 4.7 |
| Gobiesocidae | 0.397 | 0.406 | 0.677 | 7 | 2.5 |
| Gobiidae | 0.378 | 0.085 | 0.015 | 154 | 15.1 |
| Haemulidae | 0.599 | 0.084 | 0.001 | 44 | 11.7 |
| Hexagrammidae | 0.000 | 0.000 | 1.000 | 6 | 20.0 |
| Holocentridae | 0.549 | 0.137 | 0.002 | 37 | 13.6 |
| Kyphosidae | 0.631 | 0.136 | 0.375 | 31 | 20.0 |
| Labridae | 0.374 | 0.057 | 0.000 | 337 | 76.8 |
| Labrisomidae | 0.000 | 0.000 | 1.000 | 18 | 10.3 |
| Lethrinidae | 0.000 | 0.000 | 1.000 | 23 | 25.3 |
| Lutjanidae | 0.680 | 0.131 | 0.062 | 50 | 33.1 |
| Malacanthidae | 0.000 | 0.000 | 1.000 | 6 | 7.2 |
| Microdesmidae | 0.627 | 0.021 | 0.357 | 10 | 7.6 |
| Monacanthidae | 0.005 | 0.027 | 0.984 | 43 | 16.0 |
| Mugilidae | 0.005 | 0.033 | 0.998 | 6 | 2.2 |
| Mullidae | 0.016 | 0.048 | 0.959 | 29 | 14.0 |
| Muraenidae | 0.360 | 0.458 | 0.749 | 35 | 11.7 |
| Nemipteridae | 0.075 | 0.128 | 0.826 | 20 | 10.3 |
| Odacidae | 0.937 | 0.139 | 0.223 | 11 | 26.8 |
| Ostraciidae | 0.268 | 0.411 | 0.996 | 12 | 11.2 |
| Pempheridae | 0.482 | 0.136 | 0.274 | 17 | 24.6 |
| Pinguipedidae | 0.327 | 0.220 | 0.434 | 20 | 23.3 |
| Platycephalidae | 0.151 | 0.000 | 0.820 | 9 | 4.0 |
| Plesiopidae | 0.756 | 0.339 | 0.223 | 11 | 12.4 |
| Pomacanthidae | 0.524 | 0.081 | 0.012 | 42 | 22.8 |
| Pomacentridae | 0.464 | 0.074 | 0.001 | 251 | 62.4 |
| Pseudochromidae | 0.028 | 0.083 | 0.921 | 23 | 11.9 |
| Sciaenidae | 0.914 | 0.068 | 0.004 | 14 | 5.2 |
| Scombridae | 0.000 | 0.000 | 1.000 | 18 | 6.3 |
| Scorpaenidae | 0.819 | 0.120 | 0.001 | 36 | 10.2 |
| Sebastidae | 0.000 | 0.000 | 1.000 | 18 | 7.9 |
| Serranidae | 0.046 | 0.086 | 0.885 | 159 | 15.8 |
| Siganidae | 0.346 | 0.109 | 0.193 | 20 | 17.4 |
| Sparidae | 0.333 | 0.077 | 0.052 | 39 | 10.5 |
| Sphyraenidae | 0.066 | 0.168 | 0.938 | 11 | 20.0 |
| Stichaeidae | 0.000 | 0.000 | 1.000 | 8 | 4.3 |
| Synodontidae | 0.006 | 0.031 | 0.991 | 15 | 10.6 |
| Tetraodontidae | 0.442 | 0.206 | 0.154 | 40 | 21.9 |
| Tripterygiidae | 0.101 | 0.210 | 0.831 | 41 | 13.7 |
| Tree | 0.730 | 0.013 | 0.001 | - | - |

**S1 Table C. List of the ecological traits used with their nature and modalities.**

| **Trait type** | **Trait** | **Nature** | **Modalities and signification** |
| --- | --- | --- | --- |
| Body size | max_length | continuous | Maximum length of the species in cm. |
| Behavior | water_column | categorial | Position in the water column. Can be "benthic", "demersal", "pelagic site attached" or "pelagic non-site attached". |
|  | diel_activity | categorial | "day" if the species is diurnal, "night" if the species is nocturnal. |
| Feeding ecology | trophic_level | continuous | Trophic level of the species. |
|  | trophic_group | categorial | Detailed diet type. Can be "benthic invertivore", "planktivore ", "omnivore", "higher carnivore", "browsing herbivore", "excavator", "scraping herbivore", "corallivore", "algal farmer" or "cleaner". |
| Habitat use | thermal_mp_5min_95max | continuous | 5th quantile in the temperature distribution. |
|  | thermal_95thmax | continuous | 95th quantile in the temperature distribution. |
|  | habitat | categorial | Preferred habitat. Can be "sand", "coral", "rock" or "water column". |

**S1 Table D. P-values of the Tukey’s** tests indicate if there is a significant difference between the modalities of the ecological traits, habitat, water column and trophic group. p-values in bold are significant (p-values < 0.05). Data and code required to generate this Table can be found in https://github.com/nmouquet/RLS_AESTHE.

| Pair of trophic groups | p-value of Tukey test |
| --- | --- |
| Habitat | |
| coral-rock | **<0.001** |
| coral-sand | **<0.001** |
| coral-water column | **<0.001** |
| rock-sand | **0.03** |
| rock-water column | **<0.001** |
| sand-water-column | **<0.001** |
| Water column | |
| demersal-benthic | **<0.001** |
| demersal-pelagic | **<0.001** |
| benthic-pelagic | **<0.001** |
| Trophic group | |
| corallivore-excavator | 0.965 |
| corallivore-scraping herbivore | **0.021** |
| corallivore-omnivore | **<0.001** |
| corallivore-algal farmer | **0.001** |
| corallivore-cleaner | **0.003** |
| corallivore-browsing herbivore | **<0.001** |
| corallivore-benthic invertivore | **<0.001** |
| corallivore-planktivore | **<0.001** |
| corallivore-higher carnivore | **<0.001** |
| excavator-scrapping herbivore | 0.996 |
| excavator-omnivore | 0.564 |
| excavator-algal farmer | 0.819 |
| excavator-cleaner | 0.658 |
| excavator-browsing herbivore | **0.040** |
| excavator-benthic invertivore | **0.020** |
| excavator-planktivore | **0.004** |
| excavator-higher carnivore | **<0.001** |
| scraping herbivore-omnivore | 0.621 |
| scraping herbivore-algal farmer | 0.976 |
| scraping herbivore-cleaner | 0.885 |
| scraping herbivore-browsing herbivore | **0.001** |
| scraping herbivore-benthic invertivore | **<0.001** |
| scraping herbivore-planktivore | **<0.001** |
| scraping herbivore-higher carnivore | **<0.001** |
| omnivore-algal farmer | 0.999999 |
| omnivore-cleaner | 0.99999 |
| omnivore-browsing herbivore | **0.030** |
| omnivore-benthic invertivore | **0** |
| omnivore-planktivore | **0** |
| omnivore-higher carnivore | **<0.001** |
| algal farmer-cleaner | 0.9999 |
| algal farmer-browsing herbivore | 0.382 |
| algal farmer-benthic invertivore | 0.174 |
| algal farmer-planktivore | **0.026** |
| algal farmer-higher carnivore | **<0.001** |
| cleaner-browsing herbivore | 0.996 |
| cleaner-benthic invertivore | 0.983 |
| cleaner-planktivore | 0.818 |
| cleaner-higher carnivore | **0.006** |
| browsing herbivore-benthic invertivore | 0.9998 |
| browsing herbivore-planktivore | 0.541 |
| browsing herbivore-higher carnivore | **<0.001** |
| benthic invertivore-planktivore | 0.577 |
| benthic invertivore-higher carnivore | **<0.001** |
| planktivore-higher carnivore | **<0.001** |

# SI References

1. Stokes DL. Things We Like: Human Preferences among Similar Organisms and Implications for Conservation. Human Ecology. 2007;35(3):361-9. doi: 10.1007/s10745-006-9056-7.

2. Haas AF, Guibert M, Foerschner A, Co T, Calhoun S, George E, et al. Can we measure beauty? Computational evaluation of coral reef aesthetics. PeerJ. 2015;3:e1390. doi: 10.7717/peerj.1390. PubMed PMID: 26587350; PubMed Central PMCID: PMCPMC4647610.

3. Hula M, Flegr J. What flowers do we like? The influence of shape and color on the rating of flower beauty. PeerJ. 2016;4:e2106. doi: 10.7717/peerj.2106. PubMed PMID: 27330863; PubMed Central PMCID: PMCPMC4906640.

4. Tribot A, Mouquet N, Villeger S, Raymond M, Hoff F, Boissery P, et al. Taxonomic and functional diversity increase the aesthetic value of coralligenous reefs. Scientific Reports. 2016. doi: 10.1038/srep34229.

5. Graves RA, Pearson SM, Turner MG. Species richness alone does not predict cultural ecosystem service value. Proc Natl Acad Sci U S A. 2017;114(14):3774-9. doi: 10.1073/pnas.1701370114. PubMed PMID: 28320953; PubMed Central PMCID: PMCPMC5389315.

6. Tribot A-S, Carabeux Q, Deter J, Claverie T, Villeger S, Mouquet N. Confronting species aesthetics with ecological functions in coral reef fish. Scientific Reports. 2018;8. doi: 10.1038/s41598-018-29637-7. PubMed PMID: WOS:000440782000020.

7. Tribot A, Carabeux Q, Deter J, Claverie T, Villeger S, Mouquet N. Confronting species aesthetics with ecological functions of coral reef fishes. Scientific Reports. 2018;8:11733.

8. McGarigal K, Cushman SA, Ene E. FRAGSTATS v4: Spatial Pattern Analysis Program for Categorical and Continuous Maps. . University of Massachusetts, Amherst; 2012.

9. Bonhomme V, Picq S, Gaucherel C, Claude J. Momocs: Outline Analysis Using R. 2014. 2014;56(13):24. Epub 2014-01-25. doi: 10.18637/jss.v056.i13.

10. Fox J, Weisberg S. An R Companion to Applied Regression. Thousand Oaks Sage; 2018.

11. Elo A. The Rating Of Chessplayers, Past and Present: Ishi Press; 2008.

12. Clark AP, Howard KL, Woods AT, Penton-Voak IS, Neumann C. Why rate when you could compare? Using the “EloChoice” package to assess pairwise comparisons of perceived physical strength. PLoS ONE. 2018;13:1-16.

13. Tribot A, Deter J, Mouquet N. Integrating the aesthetic value of landscapes and biological diversity Proceedings of the Royal Society B. 2018;285:20180971.

14. He K, Zhang X, Ren S, Sun J, Ieee. Deep Residual Learning for Image Recognition. 2016 Ieee Conference on Computer Vision and Pattern Recognition. IEEE Conference on Computer Vision and Pattern Recognition2016. p. 770-8.

15. Deng J, Dong W, Socher R, Li L-J, Li K, Li F-F, et al. ImageNet: A Large-Scale Hierarchical Image Database. Cvpr: 2009 Ieee Conference on Computer Vision and Pattern Recognition, Vols 1-4. IEEE Conference on Computer Vision and Pattern Recognition2009. p. 248-55.

16. Cadotte MW, Jonathan Davies T. Rarest of the rare: advances in combining evolutionary distinctiveness and scarcity to inform conservation at biogeographical scales. Diversity and Distributions. 2010;16(3):376-85. doi: <https://doi.org/10.1111/j.1472-4642.2010.00650.x>.

17. Pagel M. Inferring the historical patterns of biological evolution. Nature. 1999;401(6756):877-84. doi: 10.1038/44766.

18. Blomberg SP, Garland JR. T, Ives AR. Testing for phylogenetic signal in comparative data: behavioral traits are more labile. Evolution. 2003;57(4):717-45. doi: <https://doi.org/10.1111/j.0014-3820.2003.tb00285.x>.

19. Münkemüller T, Lavergne S, Bzeznik B, Dray S, Jombart T, Schiffers K, et al. How to measure and test phylogenetic signal. Methods in Ecology and Evolution. 2012;3(4):743-56. doi: <https://doi.org/10.1111/j.2041-210X.2012.00196.x>.

20. Stuart-Smith RD, Bates AE, Lefcheck JS, Duffy JE, Baker SC, Thomson RJ, et al. Integrating abundance and functional traits reveals new global hotspots of fish diversity. Nature. 2013;501(7468):539-42. doi: 10.1038/nature12529.

21. Stekhoven DJ, Bühlmann P. MissForest—non-parametric missing value imputation for mixed-type data. Bioinformatics. 2011;28(1):112-8. doi: 10.1093/bioinformatics/btr597.

22. Iqbal H. HarisIqbal88/PlotNeuralNet v1.0.0 (Version v1.0.0). Zenodo; 2018.

23. Costello MJ, Bouchet P, Boxshall G, Fauchald K, Gordon D, Hoeksema BW, et al. Global Coordination and Standardisation in Marine Biodiversity through the World Register of Marine Species (WoRMS) and Related Databases. PLOS ONE. 2013;8(1):e51629. doi: 10.1371/journal.pone.0051629.

24. Horton T, Gofas S, Kroh A, Poore GCB, Read G, Rosenberg G, et al. Improving nomenclatural consistency: a decade of experience in the World Register of Marine Species. European Journal of Taxonomy. 2017;0(389). doi: 10.5852/ejt.2017.389.
