# Supplementary material for: The aesthetic value of reef fishes is globally mismatched to their conservation priorities
Source: PLoS Biol. 2022 Jun 7;20(6):e3001640. doi: 10.1371/journal.pbio.3001640 (PMC9173608; doi:10.1371/journal.pbio.3001640)
Supplement: Attachment — Submitted filename: Review_PBIOLOGY-D-21-02764_R1.pdf [file pbio.3001640.s006.pdf]

Review PBIOLOGY-D-21-02764_R1

Global mismatch between the aesthetic value of reef fishes and their conservation priorities

Thank you for this opportunity to review this manuscript entitled “Global mismatch between the aesthetic value of reef fishes and their conservation priorities” for Plos Biology. This manuscript presents an elegant way to evaluate the aesthetic value of reef fishes and compares this value to several attributes of the fish species, like their conservation status, their ecological traits and ecological distinctiveness. It relies on unparalleled datasets on reef fish images on one side and corresponding evaluation from the broad public (>13,000 respondents) on the other side. It thus makes for an impressive social-ecological assessment of >4,000 species of fish underpinning complementary regulating, material, and most importantly (and innovatively in the present study) non-material Nature’s Contributions to People. The paper is very well written and easy to read, and the figures nicely convey the main messages of the paper. I would like to simply congratulate the whole team of authors for their very innovative social-ecological work dedicated to find ways to quantify how human societies value their environment. I have a list of comments and suggestions that pertain to the different sections of the manuscript, that I hope will help the team improve some arguments in the introduction and discussion, and clarify a few aspects of the methods and results.

COMMENT ON THE TITLE

The title advertises an evaluation of the “global mismatches” between aesthetics and conservation threat. In the valuation literature, the term mismatch is widely used, in particular to describe the mismatches between the supply and demand for an object of valuation. In this manuscript, the term is not used in the text, neither in the introduction nor in the discussion, and having it in the title may be misleading for the readership. Besides, you also use the term “match” to describe a pair of images being compared to one another, and the term “mismatch” may also be confusing. I would suggest to slightly change the title to avoid this potential double confusion. In addition, the mismatch between the aesthetic value and the conservation status is postulated to be a concern, but this may not be the case (see my comments on the discussion).

COMMENTS ON THE INTRODUCTION

L68: what do you mean by “means of NCP” ? consider rephrasing?

L95-96: can you be a bit more specific here and explain what you mean by “visual feature”?

L.101: the sentence “to accurately identify patterns and predict values on images” is confusing. What “value” is being predicted and to what is this value assigned? As this sentence is key to describing the advantages of CNN, I would recommend to be crystal clear on what CNN does that other approaches don’t, and how it operates in more concrete terms.

L.108: “public members” feels odd (but note that I’m not a native speaker). I would suggest “13,000 respondents from the broad public”.

L.112: typo “evaluated”

L.112-119: In this paragraph, you justify the use of coral reef fishes as a study object by demonstrating their importance first for material NCP (economic activity and industry), regulating NCP (functioning of coral reef), and then only for non-material NCP (aesthetic values). I believe it would make more sense to reverse the argument, as you nicely explain in the paragraphs above that assessments of aesthetic values and, more broadly, non-material NCP are lagging behind material and regulating ones. I would therefore recommend to start with their importance in non-material terms with greater emphasis (i.e. what makes them the perfect object of study to investigate non-material NCP and aesthetic value in particular; diversity of morphologies, aesthetic values, opportunities for recreation, and probably more?), then the regulating (their contribution to the functioning of ecosystem) and then their contribution to material NCP. Besides, there is a slight contradiction between the introduction and the interpretation of the results, because reef fish species are presented as key components for economic activity (L.113-115) to justify using them as a study object, when “only” a fifth of them according to Fig.5b are used for the fishing industry. Rearranging the paragraph would restore the balance in the argumentation a bit.

COMMENTS ON THE RESULTS

Fi.1a panel (ii): the screen capture of the box capture the red underlining of Word/Powerpoint, which should be removed.

L.131-142 & L167-169: these sentences rather belong to material and methods, but I let the authors and/or the editor appreciate whether they are essential to the presentation of the results.

L.138: the term “judge” is rather unusual. I would recommend using “respondent” and stick to it throughout the text and in the supplement

L.193: specific “predicted aesthetic values”; the values have not been described so far in the text, and I believe this is an important aspect to clarify. The valuation literature is full of examples of different valuation methods, and giving a few words on the Elo method would be critical, in my humble opinion.

Fig.2a: I would suggest to have clearer labels for each of the 9 image features in the figure. The labels are rather cryptic in their current form and do not help the reading.

L212-219: why don’t you provide formal testing of the relationship between aesthetic value and both PCA axes, instead of only a visual interpretation of the patterns in Fig. 2b?

Fig.4: the color gradient should be displayed as a legend on the figure itself.

COMMENTS ON THE DISCUSSION

The paper highlights that the aesthetic value of fish is inversely proportional to their threat status and to their ecological originality/distinctiveness/usefulness (e.g.L.293). Playing the advocates' devil, isn't that a good thing that the aesthetic, intangibles values associated non-material NCP are complementary to the material and regulating NCP provided by those fish? This complementarity is an exciting finding in my opinion, as a broad spectrum of fish species are valued for complementary reasons, which makes the case for paying attention to multiple aspects to preserve them: ecological, aesthetic, and economic. To frame my question differently: what would happen if the fish species that were appreciated for their aesthetics were also highly valued by the industry, and were the most original ones and essential to the functioning of ecosystems? Worse, if they were also threatened, there would be a greater risk of an anthropogenic allee effect, whereby rare + beautiful species would suffer from an even greater demand from the broad public.

L.443-446: how is this statement backed-up by the results, and what do you mean by "less well-represented in the assessments"? I would recommend greater clarity in this statement, as it is not straightforward that data deficient species are less attractive than species present in the IUCN assessments. Besides, isn't this contradictory to the earlier statements in this paragraph about charismatic and aesthetically attractive mammal species receiving greater conservation attention (L.441-442)? Here, you demonstrate that the most aesthetically pleasing species are not particularly threatened, and will therefore not necessarily need greater conservation attention.

COMMENTS ON THE METHODS

L. 566-567: given the fact that the evaluation of the 9 features in an image is based on saturation and lightness (L.665), how did you make sure that your photoshop correction did not bias the results in any way? How many pictures did you correct that way? I do acknowledge that some corrections may have been necessary, but given the later evaluation of the image characteristics, why not discarding the images that were deemed in need of a photoshop correction?

I could not find the information on the number of "matches" undertaken by each species in the dataset. I don't doubt that with over 13,000 respondents evaluating 30 matches, the distribution of matches over all species is hypothetically even, but I was curious to know if this was the case.

It is a bit surprising that no ethical statement is provided concerning the use of a social survey. Usually, an informed consent is provided by the respondent, acknowledging that the participant undertakes the survey on a voluntary basis, that anonymity is safeguarded (this is the case here), that the respondent can opt out at no cost, and that the objective of the research has been clearly stated. Was such a consent from the participants clearly formulated?
