# Supplementary material for: The aesthetic value of reef fishes is globally mismatched to their conservation priorities
Source: PLoS Biol. 2022 Jun 7;20(6):e3001640. doi: 10.1371/journal.pbio.3001640 (PMC9173608; doi:10.1371/journal.pbio.3001640)
Supplement: Attachment — Submitted filename: RLSAESTHE_responses.pdf [file pbio.3001640.s007.pdf]

Dear Editor,

Here is our point-by-point responses to all reviewers comments (line numbers refer to the revised manuscript; codes RX#X to the reviewer X comment X).

Reviewer #1:

R1#1 : I found it an excellent and important study, considering both the implications of their results and the great potential value of the presented approach, with wide applicability in the fields of ecology, conservation, and social sciences. The manuscript is generally well developed, and the study should be relevant and of interest for a wider audience.

We thank the reviewer for this positive comment.

R1#2A : Probably the major problem in the manuscript is the fact that one part of the species were excluded from the analysis, if I understood excluded species were mainly flatfishes and seahorses. As I understood, the reason for their exclusion was that their specific morphology made them behave like outliers in the dataset that complicated the analysis? While this issue has been mostly glossed over, it is one of the main problems in the study, because it may be a source of a bias. It is important to explain and address this issue properly: 1) to provide more detailed arguments for this decision; 2) to provide exact number of species excluded from each species group; 3) to address this issue in Discussion, both in the section with caveats where it is currently not mentioned at all, as well as when discussing results. While being closer with some traits to species classified as having low aesthetic value, seahorses are considered highly attractive. I would therefore expect that they would rank highly with their aesthetic value, and they are at the same time endangered, which is an important caveat with respect to the conclusions drawn.

As mentioned in our methods, Pleuronectiformes and Syngnathiformes species were removed from the RLS database we used to homogenize the morphologies of our dataset and simplify the methodology used to estimate aesthetic values and features analysis. Pleuronectiformes represent 14 species and Syngnathiformes 31 species, which are very small proportions of the total number of species used in our survey (we use 2,417 reef fish species belonging to 139 families). We agree that seahorses (Syngnathiformes), that hold very distinct ecological traits, might be considered as more attractive by the public but their low number (14 seahorses species in the RLS database) is unlikely to change our results significatively. The flatfishes (Pleuronectiformes) also hold original ecological traits but are likely to be less attractive and thus would have almost certainly reinforced our results. Overall including these species would not have changed our main results. As requested by the reviewer, we now provide the exact number of species excluded from each species group with arguments for this decision, lines 573-578 and mention this point in our discussion lines 490-500. See also answer to comment R1#6.

R1#2B : Study included only ray-finned fishes, which is important to acknowledge better, including in the title and abstract (i.e. instead of "reef fishes" to use "ray-finned reef fishes").

For example, reef sharks are considered as highly charismatic and likely to get high aesthetic scores, while their morphology (body shape and coloration) match traits identified here as non-aesthetic.

We agree that some big fishes, such as sharks, are highly charismatic. We already mentioned the size issue in our discussion but have added a special mention to sharks lines 486-490 following the reviewer's comment. As mentioned above, given the low number of shark species (the RLS database contained 24 species of sharks), we believe this removal does not change our main results (see R1#2A). More importantly, as mentioned in our discussion, we agree that our results do not hold for a minority of species that are charismatic for reasons other than their morphology (such as sharks). We included mention to ray-finned fishes line 45 and line 110.

R1#3 : Aesthetic preferences and perceptions can considerably vary regionally, culturally, among different social and demographic groups, as well as temporally. This needs to be better addressed in the manuscript, including relevant literature, especially considering that the pool of respondents is strongly biased towards France, as well as Europe and the Global North.

We fully agree with this comment and we thought that this limitation was already discussed in our manuscript : " *We also anticipated that the results of our predictive model could be biased by individual factors, given that aesthetic preferences can vary with age, gender (Pugach et al. 2017) social groups (Kalivoda et al. 2014) or culture (Zhan et al. 2021). Nevertheless, we found no such effect. However, the absence of socio-cultural effects should be interpreted with caution, because of the relatively limited diversity of socio-cultural backgrounds of our respondents. For example, 62.4% of the judges were French and 84.9% had a Bachelor's degree or a higher diploma (Text C in S1 File). (...) We might have found some differences if we had compared populations with very different cultural backgrounds or asked questions that would have triggered cognitive processing; which opens interesting perspectives for future research*". We have tried to strengthen this paragraph and added a reference to the recent framework of "personalised ecology" proposed by Gaston et al. (Gaston, K. J. 2020. Personalised ecology and detection functions. People and Nature 2:995-1005.), lines 507-530.

R1#4: There is insufficient information about how the questionnaire was distributed, whether there were financial or other incentives to distribute it, and especially the languages in which it was provided, to understand the reasons for such strongly biased pool of respondents. I suspect based on information in Lines 491-492 that the questionnaire dissemination was improperly planned, aiming mainly to respondents in France, and using academic or similar mailing lists, which resulted in such skewed pool of respondents, both spatially and with respect to education level, mostly failing to reach other countries, continents and regions, as well as general population, beyond the highly educated respondents. This is one of the major weaknesses of the study, and needs to be better acknowledged and discussed in the paper.

The questionnaire was available in French and English. We added this information and more details on how the questionnaire was distributed in the method section (lines 643-648). The reviewer is right that the dissemination was biased toward the French public which resulted

from our better knowledge of the French networks. We have nevertheless disseminated heavily toward English speaking public through social media and emailing to the major universities and conservation societies abroad. We agree that we obtained a skewed pool of respondents, both spatially (64% are French) and with respect to education level (84.9% had a Bachelor's degree or a higher diploma). Note however that French respondents can be culturally different and geographically widespread given the number of overseas territories across all the oceans. But despite this geographical and education biases we found no effect of geographic or socio-cultural backgrounds when analyzing the robustness of the matches outcomes (probability for an image to win a match) which indicates that our results are robust to this type of respondent bias. We highlight this critical point lines 168-173, lines 643-648 and lines 507-530.

R1#5: Authors have pooled species that are categorized by the IUCN Red List as Not Evaluated (NE) and Data Deficient (DD) into a single group for analyses, "Not Evaluated". However, there is a considerable literature showing that Data Deficient species tend to be overall highly threatened (i.e., their traits, low population size, small ranges, and other characteristics that are making them highly threatened are also making them poorly studied and consequently data deficient). For this reason, DD species do not really belong together with NE, especially considering that comparisons of NE and TH groups were used to conclude that threatened species have lower aesthetic value. I do not think or suggest that it is necessary to redo complete analysis with DD species considered separately, but authors should at least acknowledge this issue and refer to the literature that addressed the issue of the threat status of data deficient species.

The Data Deficient species are not frequent in our dataset (n=69). Given the reviewer comment, however, we decided to remove them from our analysis of IUCN status to avoid any confusion on the result we found with the Not Evaluated species which are much more common(n= 556). We prefer not to consider that the Data Deficient species tend to be highly threatened as we could not find any clear evidence for this in the fish conservation literature. We have redone our analysis and figures (both figure 5 and figure S in S1 File) and modified our main text accordingly. Removing the 69 Data Deficient species has not changed our results. Note that we have re-extracted up-to-date IUCN status from Fishbase which has resulted in a slight modification of the numbers of species TH (+1 species) and LC (+25 species) without any consequences on our first results.

R1#6: It would be good to include a table, at least in the supplementary material, which would present number of species and photos from different sources, used in each step (e.g. initial number of species, species excluded, etc.), to make it easier to understand the dataset used.

This table is now provided (S2 Data) and mentioned in the methods (line 584). The list of photos is provided in table S4 Data.

R1#7: Referring to respondents of the questionnaire as "judges" makes those sections confusing. It would be clearer and more appropriate to refer to them as "respondents".

We now refer only to respondents in the main text and in the supplementary material.

R1#8: There is some repetition in Results section of the information that is already presented in Methods section. Most of the subchapters in Results begin with description of methods. Such sections should be deleted, or at least condensed to reduce repetitiveness of the text.

In our revised manuscript, we have reduced the description of the methods in the results section when possible.

R1#9: . Line 132, "Figure 1A" - "A" shouldn't be capitalized

We refer to figures following the PlosBiology requirement for figures In-text citations : “Cite figures with the format: Fig 1A, Fig 1B, Fig 2, Fig 3, etc.”
https://journals.plos.org/plosbiology/s/figures

R1#10:. It is unnecessary to refer to names of variables that were used during the analysis (e.g. "CL_cie_d_mean"), these names are not relevant and make it more difficult to follow the text (e.g. in Lines 225-228 and in Figure 2). Instead of those names it would be better to simply refer to the actual variable (e.g. "color heterogeneity").

We understand that the variable names might be misleading and have tried to come up with more meaningful names. This has been done only for the 9 selected variables that explained a significant proportion of the variation in the aesthetic values. We have modified the main text, the Fig. 2A and B and the text F in S1 accordingly.

R1#11: Figure 3 caption (Line 269) mentions "ecological distinctiveness", while the X axis in the figure is named "Functional distinctiveness". Terms should be consistently used in the study.

We now use ‘functional distinctiveness” in the whole manuscript.

R1#12. Some species names in Figure 4 caption appear twice (Pterois miles and Cephalopholis sexmaculata in Lines 283-284). Just in case, please check this also in other parts of the manuscript, figures and supplementary material, as well as spelling of all species names, as it is one of common source of errors.

Thanks for noticing this, we corrected the Fig. 4 caption. We have checked all figure captions in the main text and supplementary material.

R1#13. Line 324 - "Least Concerned" should be corrected to "Least Concern"

Done

R1#14. Line 400 - "though" should be corrected to "through"

Done

R1#15. Line 545 - it is left unclear why were these 137 species from FishBase not already covered by the original dataset of 2280 species, why were they missing from RLS?

These species were not observed in RLS surveys. We made this point more clear lines 581-582.

R1#16.. Line 593, "Fig S5" - Which figure is this? Figures in the supplementary material are all named with capital letters.

We were referring to Fig E in S1 file. Thanks for having spotted this error.

R1#17. Line 609 - "images" should be in singular

Done.

R1#18. Line 763 - Sentence should be corrected to "Data on IUCN threat status are available in the IUCN Red List database".

Done.

R1#19. S1 Fig G - legend of the inset figure needs more values on the color scale, not just the maximum and minimum. It should also not be log transformed, as it does not present clearly bias in spatial coverage.

Done.

R1#20. S1 Fig. H - Y axis categories need to be made clear (e.g. education, place, distance, etc.)

We made the Y axis clearer and detailed the modalities in the figure legend.

Reviewer #2:

R2#1 : As conservation, public/political, and research attention is greatly affected by aesthetic qualities of biota, this research is highly important and stands to make a large impact.

We thank the reviewer for this tribute.

R2#2: I have a concern about the representativeness of the population of people surveyed. The paper addresses the aesthetic value of global coral reef fishes, and does so by distributing online surveys through existing networks. Does this create a cultural bias in the sampled population with respect to the global population, perhaps especially neglecting those people interacting with coral reef fish through subsistence fishing or other livelihoods? Furthermore, it is indicated that the selection of fish species ensured that they were those encountered by divers, but the diving theme is not further developed or linked to the sampled population. Generally I would like to see more consideration and discussion of the rationale and global representativeness of the sampled population, and a more explicit explanation of

whether the focus is on fish encountered by SCUBA divers (as hinted at), and therefore perhaps a population of potential tourist SCUBA divers, or whether the study and results are meant to be more broadly applicable across populations around the world and with respect to different ways in which people worldwide interact with coral reef fish, including in subsistence settings where some of the highest rated fishes are fished.

We now provide more detail in the method on the biases associated with our dissemination strategy (lines 643-648). The reviewer is right that the dissemination was biased which resulted in a skewed pool of respondents, both geographically (64% are French) and with respect to education level (84.9% had a Bachelor's degree or a higher diploma) as well as SCUBA diving (52% had an experience with scuba diving; we added mention to this % line 516). Note however that French respondents can be culturally different and geographically widespread given the number of overseas territories across all the oceans. But despite these biases we found no effect of respondents' geographic origin or backgrounds when analyzing the robustness of the matches outcomes (probability for an image to win or not a match) which indicates that our results are robust to these respondent biases. For instance, images have the same probability of winning a match if they are judged by respondents with experience with scuba diving or not. We added mention to the SCUBA divers in the discussion line 516 and already discussed the representativeness of the population of people surveyed lines 507-530.

JL R2#3: Generally the AI algorithm does well at predicting aesthetic scores (Fig. 1B). However, in perhaps the top 5 or 10% of the range of evaluated aesthetic scores the algorithm does much more poorly. After X = 1750 all of the points are above the line, meaning AI was over-estimating the aesthetic values. I don't expect this to influence the results (looking at the other figures) but checking these points don't change the later models might be useful.

Fig 1B represents only the five sets used to perform the cross-validation (each set containing 93 +/- 3.6 species) which are used to compute the performance of our AI algorithm. To do so we merged the five cross-validation prediction sets and computed the linear regression to provide a r^2 (see lines 674-677*)*. If you look at each cross-validation set (see new figure S1 M and S1 text E) you can see that none (except maybe set 4) overestimates the aesthetic value of images for the highest scores. The final predictions have been obtained by training the model one more time with all images and we used these weights to make our final predictions. To make this point even clearer we now present the regression between the predicted and evaluated values for the whole learning set (481 images) on figure S1 M (see also S1 text E) which clearly shows that our final predictive model does not overestimate the aesthetic values of the highest scores. We have modified our S1 Text E section to make this point clear and added one new figure (S1 Fig. M).

R2#4: The statistical models relating aesthetic value with species age, ED and functional distinctiveness do not appear to account for phylogenetic relatedness, even though aesthetic value shows a strong phylogenetic signal. I realize that it is not always relevant to account for phylogeny in functional analyses, but it would be useful if the authors could comment on the rationale for leaving out phylogeny from the functional analysis, and what the implications are regarding inference? Does this limit our approach to conclude whether the differences are being driven by a couple of key contrasting families (e.g., beautiful

butterflyfishes vs. brutish barracudas?), as opposed to a more general effect across the Tree?

We agree with the reviewer that the relationship between aesthetic value with species age, ED and functional distinctiveness is somewhere linked to phylogenetic relatedness as expected from the strong phylogenetic signal we found for the aesthetic value. To account for phylogenetic relatedness we added a model that takes into account the phylogenetic distance between species pairs at the fitting stage using the phylolm() function of the phylolm R package, using each of the 100 trees used in our study. We found that the three relationships remained significant when accounting for phylogenetic relatedness. The new results are given lines 260-265. We have modified the methods accordingly lines 794-799. The figures 3 has been redrawn, as well as the Fig N in S1 showing the two relationships.

R2#5: Red squiggly spell-check lines in 1A should be removed

Done, thanks for noticing this.

Reviewer #3:

[identifies himself as Uri Roll]

R3#1 : I found this contribution to be interesting, novel, and important. I have but few minor comments to it.

We thank the reviewer for this positive comment.

R3#2: I thought that the abstract could benefit from specifically mentioning that you used fishes' images (perhaps also mention from readily available online sources)

We now mention that we used fish images in the abstract line 46.

R3#3: Perhaps mention in the methods the socio-cultural variables you explored even broadly (and not just in the supp.)

Done lines 640-642.

R3#4: In figure 2 try to give the actual names of features and not their abbreviations.

We understand that the variable names might be misleading and have tried to come up with more meaningful names. This was done only for the 9 selected variables that explained a significant proportion of the variation in the aesthetic value. We have modified the main text, the Fig. 2A and B and the text F in S1 accordingly.

R3#5: Generally, try to reduce a bit the repetition between the methods and results section.

In our revised manuscript, we have reduced the description of the methods in the results when possible.

Reviewer #4:

R4#1 : The paper is very well written and easy to read, and the figures nicely convey the main messages of the paper. I would like to simply congratulate the whole team of authors for their very innovative social-ecological work dedicated to find ways to quantify how human societies value their environment.

We thank the reviewer for this positive comment.

R4#2: The title advertises an evaluation of the "global mismatches" between aesthetics and conservation threat. In the valuation literature, the term mismatch is widely used, in particular to describe the mismatches between the supply and demand for an object of valuation. In this manuscript, the term is not used in the text, neither in the introduction nor in the discussion, and having it in the title may be misleading for the readership. Besides, you also use the term "match" to describe a pair of images being compared to one another, and the term "mismatch" may also be confusing. I would suggest to slightly change the title to avoid this potential double confusion. In addition, the mismatch between the aesthetic value and the conservation status is postulated to be a concern, but this may not be the case (see my comments on the discussion).

We would like to keep the term "*mismatch*" in our title as it clearly highlights our main finding that “*The most beautiful fishes are tightly packed into small regions of both the phylogenetic tree and the ecological trait space. In contrast the less attractive fishes are the most ecologically and evolutionary distinct species and those recognized as threatened*”. The term "*mismatch*" was already used in our abstract and we now use it in our discussion line 342 and line 450 to mirror the title of our ms.

R4#3: L68: what do you mean by "means of NCP" ? consider rephrasing?

We have rephrased it to “most other NCP” line 70.

R4#5: L95-96: can you be a bit more specific here and explain what you mean by "visual feature"?

We have rephrased it to “... use visual features (i.e. pattern analysis, color distribution) in images known…” lines 97-98.

R4#6 L.101: the sentence "to accurately identify patterns and predict values on images" is confusing. What "value" is being predicted and to what is this value assigned? As this sentence is key to describing the advantages of CNN, I would recommend to be crystal clear on what CNN does that other approaches don't, and how it operates in more concrete terms.

We have rephrased it to “ …to accurately identify patterns (classification tasks) and predict continuous variables on images” lines 103-104.

R4#7: L.108: "public members" feels odd (but note that I'm not a native speaker). I would suggest "13,000 respondents from the broad public".

Done.

R4#8: L.112: typo "evaluated"

Done.

R4#9: L.112-119: In this paragraph, you justify the use of coral reef fishes as a study object by demonstrating their importance first for material NCP (economic activity and industry), regulating NCP (functioning of coral reef), and then only for non-material NCP (aesthetic values). I believe it would make more sense to reverse the argument, as you nicely explain in the paragraphs above that assessments of aesthetic values and, more broadly, non-material NCP are lagging behind material and regulating ones. I would therefore recommend to start with their importance in non-material terms with greater emphasis (i.e. what makes them the perfect object of study to investigate non-material NCP and aesthetic value in particular; diversity of morphologies, aesthetic values, opportunities for recreation, and probably more?), then the regulating (their contribution to the functioning of ecosystem) and then their contribution to material NCP. Besides, there is a slight contradiction between the introduction and the interpretation of the results, because reef fish species are presented as key components for economic activity (L.113-115) to justify using them as a study object, when "only" a fifth of them according to Fig.5b are used for the fishing industry. Rearranging the paragraph would restore the balance in the argumentation a bit.

Thanks for the suggestion, we have reversed the argument as suggested lines 116-123.

R4#10: Fi.1a panel (ii): the screen capture of the box capture the red underlining of Word/Powerpoint, which should be removed.

Done, thanks for noticing this.

R4#11: L.131-142 & L167-169: these sentences rather belong to material and methods, but I let the authors and/or the editor appreciate whether they are essential to the presentation of the results.

We have reduced the description of the methods in the results when possible. We hope the reviewer and editor will find this section more fluid.

R4#12: L.138: the term "judge" is rather unusual. I would recommend using "respondent" and stick to it throughout the text and in the supplement

Done. Thanks for the suggestion.

R4#13: L.193: specific "predicted aesthetic values"; the values have not been described so far in the text, and I believe this is an important aspect to clarify. The valuation literature is

full of examples of different valuation methods, and giving a few words on the Elo method would be critical, in my humble opinion.

We could not find the specific point mentioned by the reviewer L.193. We believe the reviewer mentioned the sentence L141-142 : “This method provided a robust estimation of fish aesthetic value through Elo scores computation [30].” . As requested by the reviewer (R4#11) and two other reviewers, we will keep the method description to the minimum in the results section and thus will refer here to the method where more information is given on how we computed aesthetic values and used the Elo score. The sentence now reads “We then estimated each fish aesthetic value through Elo scores computation (see methods)” lines 142-143.

R4#14: Fig.2a: I would suggest to have clearer labels for each of the 9 image features in the figure. The labels are rather cryptic in their current form and do not help the reading.

We understand that the variable names might be ambiguous sometimes so we have tried to come up with more descriptive names. This has been done only for the 9 selected variables that explained a high proportion of variation in aesthetic value. We have modified the main text, the Fig. 2A and B and the text F in S1 accordingly.

R4#15: L212-219: why don't you provide formal testing of the relationship between aesthetic value and both PCA axes, instead of only a visual interpretation of the patterns in Fig. 2b?

We are not sure what the reviewer means by a “formal testing of the relationship between aesthetic value and both PCA axes”. The formal test of the relationship between aesthetic and features is provided in Fig. 2A and associated analysis. The Fig. 2B is only used to project image features on the two first synthetic PCA axes and illustrates the result presented in Fig. 2A. with some archetypal fishes. Using the two first axes of the PCA is a simplified visualization but cannot be used in formal testing.

R4#16: Fig.4: the color gradient should be displayed as a legend on the figure itself.

Done.

R4#17: The paper highlights that the aesthetic value of fish is inversely proportional to their threat status and to their ecological originality/distinctiveness/usefulness (e.g.L.293). Playing the advocates' devil, isn't that a good thing that the aesthetic, intangibles values associated non-material NCP are complementary to the material and regulating NCP provided by those fish? This complementarity is an exciting finding in my opinion, as a broad spectrum of fish species are valued for complementary reasons, which makes the case for paying attention to multiple aspects to preserve them: ecological, aesthetic, and economic. To frame my question differently: what would happen if the fish species that were appreciated for their aesthetics were also highly valued by the industry, and were the most original ones and essential to the functioning of ecosystems? Worse, if they were also threatened, there would be a greater risk of an anthropogenic allee effect, whereby rare + beautiful species would suffer from an even greater demand from the broad public.

We thank the reviewer for this very interesting comment. Our main point here is that the implication of aesthetic value for biodiversity conservation/research effort is substantial; the mismatch we found between aesthetic value, r threat status and ecological distinctiveness is an issue. To illustrate this point we have defined an “aesthetic debt” that the less beautiful species would pay: they get potentially less attention/research/conservation efforts, are more exploited, while they are more important to ecosystem functioning than the more beautiful species (at least they provide more distinct functions). We agree, however, that the result of aesthetic value being inversely proportional to ecological distinctiveness and economic importance (at least fishery importance) provides a good argument for making aesthetic value a complementary facet of biodiversity, compared to the most common ecological and economic facets. We have added a sentence at the beginning of our conclusion to make this point clearer (largely inspired by the reviewer comment) which echoes also with the comment R4#2 on using the term “mismatches”, lines 531-539.

R4#18: L.443-446: how is this statement backed-up by the results, and what do you mean by "less well-represented in the assessments"? I would recommend greater clarity in this statement, as it is not straightforward that data deficient species are less attractive than species present in the IUCN assessments. Besides, isn't this contradictory to the earlier statements in this paragraph about charismatic and aesthetically attractive mammal species receiving greater conservation attention (L.441-442)? Here, you demonstrate that the most aesthetically pleasing species are not particularly threatened, and will therefore not necessarily need greater conservation attention.

We thank the reviewer for noticing that this statement missed a proper test. To make this result straightforward we should have combined the species in the Threatened and the Least Concern categories into an “Evaluated” category and compared it with the “Not Evaluated” category. We have performed this analysis and found a significant effect ($p<0.001$) which means that overall fishes evaluated for the IUCN Red List have higher aesthetic values than the not evaluated fishes. We added this in our result section lines 311-314. We don’t think this result is contradictory to the earlier statements about mammals, which related to research effort rather than conservation attention (specifically in relation to the work by Fleming and collaborators 2016).

R4#19: L. 566-567: given the fact that the evaluation of the 9 features in an image is based on saturation and lightness (L.665), how did you make sure that your photoshop correction did not bias the results in any way? How many pictures did you correct that way? I do acknowledge that some corrections may have been necessary, but given the later evaluation of the image characteristics, why not discarding the images that were deemed in need of a photoshop correction?

Finding and preparing images for 2,417 fishes have been a long process. We gathered 4,881 images, and for each we had to remove the natural background and standardize fish position. For some species there were numerous images available and we could choose good quality images. In some other cases, few images were available and we had to correct for poor luminosity and oversaturation (in the blue and the green essentially). Photoshop provides built-in functions to correct these issues. We believe that these corrections did not influence our results as the effect of fish features on the aesthetic values comes from extreme features (as shown in Fig. 2B) and not only saturation and lightness. All the images

have been processed with Photoshop at least for cropping and to standardize the position and size of fishes; the luminosity and saturation correction was decided on a case by case basis without taking record of exact modification. Overall our results are very consistent among fishes as illustrated by the few images (for which we had copyrights) we have chosen to illustrate the figures 1, 2 and 3. More generally, Pomacanthidae and Chaetodontidae are perceived as more beautiful than Gerreidae or Scombridae which makes sense so we believe the correction we made for poor luminosity and oversaturation did not influence our results. We added details on the saturation correction lines 602-604.

R4#20: I could not find the information on the number of "matches" undertaken by each species in the dataset. I don't doubt that with over 13,000 respondents evaluating 30 matches, the distribution of matches over all species is hypothetically even, but I was curious to know if this was the case.

Yes, given the high number of matches, the distribution of the matches over all images should be even. We have calculated the average number of matches per image. In doing so, thanks to the reviewer comment, we realized that the total number of matches we were providing in the methods was wrong (it resulted from our initial survey containing more images than the 345 images used in our training sets; these extra images were actually concerning species not present in the RLS database and were not considered in this study). This does not change any of our results as all analyses are performed with the right number of images and matches (we checked this thoroughly). To summarize our final numbers, the total number of matches analyzed was 289,650; as each match contains two images that makes 289,650 x 2 = 579,300 images judged which should lead to 1,678 matches per image. We have looked at the distribution of matches per image which is very tight (mean 1,679 +/- 40) which confirms that the number of respondents was enough to reach the expected even number of matches per image. We modified the text D in S1 to provide this number : “Given the high number of respondents (13,000) the distribution of the matches per species was even (mean 1,679 +/- 40).”

R4#21: It is a bit surprising that no ethical statement is provided concerning the use of a social survey. Usually, an informed consent is provided by the respondent, acknowledging that the participant undertakes the survey on a voluntary basis, that anonymity is safeguarded (this is the case here), that the respondent can opt out at no cost, and that the objective of the research has been clearly stated. Was such a consent from the participants clearly formulated?

The internet questionnaire we used contained an introduction that clearly stated the objective of the research and an ethical statement to guarantee anonymity to the respondents; we provided each respondent with an anonymity identification number which could be used to opt out at no cost following the *French* Data Protection Authority (*CNIL*) recommendation. We added this information in the S1 Text C. We are not sure this implies adding an ethics statement in the method section (as mentioned on the Plos submission site) and let the editor decide if we need to add this section in our main text.
